# How Does the Bonding Strength of Reline Materials and Denture Teeth Vary Between 3D‐Printed and Milled Complete Denture Bases? A Systematic Review and Meta‐Analysis

**DOI:** 10.1002/cre2.70234

**Published:** 2025-10-07

**Authors:** Sarah Arzani, Erfan Khorasani, Aida Mokhlesi, Shima Azadian, Safoura Ghodsi, Seyed Ali Mosaddad

**Affiliations:** ^1^ Child Growth and Development Research Center, Research Institute for Primordial Prevention of Non‐Communicable Disease Isfahan University of Medical Sciences Isfahan Iran; ^2^ Research Committee Qazvin University of Medical Sciences Qazvin Iran; ^3^ USERN Office Qazvin University of Medical Sciences Qazvin Iran; ^4^ Social Determinants of Health Research Center, Research, Institute for Prevention of Non‐Communicable Diseases Qazvin University of Medical Sciences Qazvin Iran; ^5^ Department of Biostatistics Shiraz University of Medical Sciences Shiraz Iran; ^6^ Department of Prosthodontics Dental Research Center, Dentistry Research Institute, Tehran University of Medical Sciences Tehran Iran; ^7^ Department of Research Analytics Saveetha Dental College and Hospitals, Saveetha Institute of Medical and Technical Sciences, Saveetha University Chennai India; ^8^ Department of Conservative Dentistry and Bucofacial Prosthesis Faculty of Odontology, Complutense University of Madrid Madrid Spain; ^9^ Department of Prosthodontics School of Dentistry, Shiraz University of Medical Sciences Shiraz Iran

**Keywords:** computer‐aided design, dental bonding, dental materials, denture bases, printing, three‐dimensional

## Abstract

**Objective:**

To systematically compare the bond strength of denture teeth and reline materials to additively manufactured (AM) versus subtractively milled (SM) denture base resins and to identify the material‐ and process‐related factors influencing bonding performance.

**Materials and Methods:**

A systematic electronic search of PubMed, Scopus, Embase, Web of Science, the Cochrane Library, and Google Scholar was conducted up to December 10, 2024. Eligible in vitro studies comparing bond strength at either the tooth–base or reline–base interface using AM and SM denture bases were included. Studies that lacked direct comparison, involved conventional heat‐polymerized bases, or did not report quantitative bond strength data were excluded. Meta‐analyses were performed using random‐effects models, calculating mean differences (MD) for tooth bonding and standardized mean differences (SMD) for reline bonding. Subgroup, sensitivity, and publication bias analyses (Egger's regression and Begg's rank tests) were included. Risk of bias was evaluated using QUIN tools.

**Results:**

Out of 2985 screened records, 20 studies comprising 156 independent comparisons were included; 41 for tooth bonding and 115 for reline bonding. Initial tooth‐bonding meta‐analysis revealed no significant difference; however, after exclusion of two outlier comparisons identified through sensitivity analysis (*n* = 39), milled bases demonstrated significantly higher bond strength (MD = −2.43 MPa, 95% CI–3.90 to −0.96; *p* = 0.001). For reline bonding, AM bases consistently underperformed across all studies, with the pooled estimate favoring milled bases (SMD = −2.62, 95% CI–3.22 to −2.03; *p* = 0.001).

**Conclusion:**

Within the limitations of this review, milled denture bases demonstrate consistently stronger and more reliable bonding to both teeth and reline materials than current printable photopolymer bases.

## Summary

- These findings indicate that subtractively milled denture bases provide more durable and predictable bonding to denture teeth and reline materials than current additively manufactured bases.
- Clinically, this suggests that milled dentures remain the safer choice when long‐term retention and reliable relining are required, whereas printed dentures may necessitate more frequent maintenance and repairs.

## 1 Introduction

Edentulism remains a prevalent chronic condition that compromises mastication, phonetics, and facial support in affected individuals (Niakan et al. 2024). Conventional heat‐polymerized polymethyl methacrylate (PMMA) complete dentures have long been a cost‐effective and clinically successful solution for these patients (Brígido et al. 2023; Jar et al. 2023). Nonetheless, conventional techniques are limited by polymerization shrinkage, porosity, residual monomer release, and technique sensitivity, all of which can undermine long‐term durability (Abualsaud and Gad 2022; Ayman 2017; de Oliveira Limírio et al. 2022).

The push to overcome these shortcomings has paralleled the rapid evolution of digital dentistry. Subtractive computer‐aided design/computer‐aided manufacturing (CAD/CAM) milling (SM) and, more recently, additive manufacturing (AM), also known as three‐dimensional (3D) printing, allow laboratory steps to be standardized, accelerated, and, in many cases, automated (Dusmukhamedov et al. 2021; El Osta et al. 2025; Swapna 2024). Milled dentures are carved from highly dense, pre‐polymerized PMMA discs with minimal residual monomer, high flexural strength, and low porosity; yet they incur significant material waste, require expensive multi‐axis mills, and offer limited aesthetic and shade‐layering options (Khorasani et al. 2025; Srinivasan et al. 2018; Srinivasan et al. 2021). By contrast, AM builds dentures layer‐by‐layer from photo‐curable resins with virtually no waste and unparalleled design freedom (Agrawaal and Thompson 2021); however, incomplete cross‐linking, volumetric shrinkage, staircase surface roughness, and anisotropic mechanical properties have raised legitimate concerns about structural integrity (Ngo et al. 2018; Rooney et al. 2024). Moreover, variability in printing technologies, differences in resin formulations, and inconsistent postprocessing protocols further complicate efforts to standardize bonding outcomes (Rooney et al. 2024), while additional workflow‐specific drawbacks—elimination of the conventional try‐in appointment without a fully validated virtual aesthetic check, poor intrinsic retention of printed polymers that often necessitates chairside relining, difficulty achieving balanced occlusion (with attendant risks for diminished denture stability and accelerated ridge resorption), and long‐term color instability that compromises aesthetics—continue to limit widespread clinical adoption (Anadioti et al. 2020).

Since neither milling nor printing can directly fabricate multi‐shade denture teeth in situ, secondary bonding of prefabricated teeth to the digital base remains mandatory. The strength of this interface is critical: failed tooth bonds lead to fracture, aesthetic loss, and costly repairs (Löscher et al. 2024; A. Mohamed et al. 2023a). Bond durability depends on resin chemistry, surface energy, pretreatment, and the compatibility of bonding agents (Dimitrova et al. 2024; Löscher et al. 2024). Similarly, chairside relining or laboratory re‐basing is often required to compensate for ongoing ridge resorption and improve denture fit; reliable base‐to‐reline adhesion is therefore a second cornerstone of long‐term clinical success (Singla 2022). Yet, the distinct surface morphology and polymer networks created by digital workflows may compromise chemical diffusion and mechanical interlocking, jeopardizing bond performance (Hammer et al. 2021).

Despite a growing body of in vitro data (Alanazi et al. 2024; Albazroun et al. 2024; Alfaraj et al. 2023; Awad et al. 2023; Choi et al. 2020; Gad et al. 2022; Gibreel et al. 2024; Htat et al. 2024; Janyaprasert et al. 2024; Kane and Shah 2023; Karaokutan and Ayvaz 2025; Karaokutan et al. 2024; Li et al. 2025; Löscher et al. 2024; Mert et al. 2023; A. Mohamed et al. 2023a; Sahin et al. 2024b; Tugut et al. 2024; Vuksic et al. 2023; Wemken et al. 2021), no consensus has emerged on whether SM or AM bases provide superior bonding to denture teeth and reline materials nor on which processing variables most strongly modulate that performance. Given the distinct fabrication‐ and material‐related variables (Dimitrova et al. 2024; Löscher et al. 2024), a thorough investigation into how these digital methods influence the bonding strength of denture components is essential for guiding evidence‐based clinical practice. Most prior reviews have centered chiefly on clinical outcomes (Alhallak et al. 2023; Alotaibi 2025; Avelino et al. 2024; Khorasani et al. 2025; Zandinejad et al. 2024); although one narrative review addressed bonding performance (Tzanakakis et al. 2023), it was not systematic. No previous systematic review has simultaneously examined both denture tooth–base and reline–base adhesion across the spectrum of digital fabrication technologies, which underscores the need for further investigation to provide comprehensive evidence on this subject. Therefore, the present systematic review and meta‐analysis aimed to compare the bond strength of denture teeth and reline materials to AM versus SM complete denture bases. The null hypothesis was that digitally milled and 3D‐printed dentures would exhibit no statistically significant differences in bonding performance at either interface.

## 2 Materials and Methods

### 2.1 Study Design and Primary Research Question

This study followed the PRISMA guidelines (Preferred Reporting Items for Systematic Reviews and Meta‐Analyses) along with the recommendations outlined in the Cochrane Handbook for Systematic Reviews (Page et al. 2021). The study protocol was registered on the Open Science Framework (OSF) and can be accessed via the https://doi.org/10.17605/OSF.IO/3MSZY. Table 1 presents the PICO framework (Population, Intervention, Comparison, and Outcome), which guided the formulation of the primary research question: “How does the bonding strength of reline materials and denture teeth compare between 3D‐printed and milled complete denture bases?”

**Table 1:** Detailed description of the population, intervention, comparison, and outcome (PICO) criteria guiding the study design.

PICO elements	PICO‐based study design
P	Digitally fabricated complete denture bases, either in complete denture form or as individual specimens
I	Additively manufactured (3D‐printed) denture bases
C	Subtractively manufactured (milled) denture bases
P	Denture teeth‐ and reline material‐denture base bond strength

### 2.2 Databases and Search Strategy

To gather relevant studies, a comprehensive search was performed across five major databases: PubMed (Medline), Embase, Scopus, Web of Science, and the Cochrane Library. The search spanned up to December 10, 2024, with a focus on English‐language studies but without restrictions on publication dates. The search strategy, outlined in Table S1, combined free‐text keywords with controlled vocabulary terms, such as MeSH and Emtree, to enhance retrieval accuracy. Additionally, reference lists of selected and related articles were manually reviewed, and a supporting search on Google Scholar was conducted to identify any overlooked publications. All retrieved records were systematically organized and prepared for screening using EndNote 21 (Clarivate Analytics).

### 2.3 Eligibility Criteria

The inclusion criteria were limited to in vitro studies published in English in peer‐reviewed journals that investigated the bonding strength of reline materials and denture teeth to digitally fabricated complete denture bases. Eligible studies specifically measured bond strength using shear or tensile methods and directly compared 3D‐printed and milled denture bases. Exclusion criteria included studies that evaluated bond strength to denture bases fabricated using hybrid approaches that combined conventional and digital techniques or assessed the bonding of repair resins for other types of prostheses. Modified resins incorporating additional elements were also outside the scope of this analysis. Additionally, the following study designs and formats were excluded: clinical studies, animal studies, observational studies, pilot studies, case reports, case series, systematic reviews, meta‐analyses, narrative reviews, finite element analyses, technical reports, short communications, letters, commentaries, editorials, conference abstracts, book chapters, studies lacking a control group, and studies with incomplete data.

### 2.4 Study Selection and Data Extraction

The selection process was conducted in two stages using EndNote 21 (Clarivate Analytics) to manage references and identify duplicate records, which were removed manually. In the first stage, two reviewers (S. A. and E. K.) independently screened the titles and abstracts of all retrieved studies in a blinded manner. In the second stage, the full texts of the remaining studies were assessed against the predefined inclusion and exclusion criteria to determine final eligibility. Before starting the evaluation, the reviewers underwent calibration by screening a random sample comprising 10% of the studies. The inter‐rater reliability during both stages of screening was measured using Cohen's Kappa coefficient (*κ*). The *κ* values for the first and second screening stages were 0.89 and 0.95, respectively, indicating a high level of inter‐rater agreement. Any discrepancies during the screening process were resolved through mutual consensus between the two reviewers or, if needed, by involving a third author (S. A. M.).

Afterward, the same two reviewers independently extracted relevant information from the included studies using a standardized Excel form. The data collected included general study characteristics such as the first author, year of publication, and country, as well as specific study design details, including the study model, digitization method, scanning device, and design software. For studies in the AM group, extracted data included the printer device model and manufacturer, printing technology, material type and manufacturer, layer thickness, build orientation, specimen type, dimensions, shape, and post‐processing procedures. Similarly, for the SM group, information was collected on the milling machine brand, number of axes, material type and brand, specimen type, dimensions, shape, and postprocessing procedures. Specific information was also gathered on tooth‐base bonding, including the material and manufacturer of the bonded teeth, chemical composition, tooth dimensions and shape, bonding agent used, surface treatments applied to the teeth, and aging procedures. Regarding reline material–base bonding, the data extracted covered liner specimen dimensions, liner type and polymerization method, liner material and manufacturer, denture base surface preparation, bonding agent used, and any treatments performed before bond strength testing. Additional data included sample size, evaluation methods, and statistical outcomes. In studies assessing multiple parameters of interest, each parameter was treated as a separate entry in the analysis. When necessary, missing information was retrieved by contacting the corresponding authors of the original articles. Moreover, when results were presented only in graphical form, numerical values were extracted using PlotDigitizer software version 3.3.9 PRO (PlotDigitizer.com, USA) independently by two reviewers (S. A. and S. A.) and double‐checked by the statistical specialist who conducted the meta‐analyses. Any discrepancies during the data extraction process were resolved through discussion with a fourth author (S. G.).

### 2.5 Statistical Analyses

This value, calculated based on the frequency of exact agreements between the two reviewers, required a minimum threshold of 80% agreement to be considered acceptable. Two separate meta‐analyses were conducted. The first focused on studies reporting the bond strength between denture teeth and denture bases, while the second analyzed the bond strength between denture bases and reline materials. When a single study contributed multiple effect sizes, these were treated as independent comparisons and included in the random‐effects model, which accounts for between‐study variance. For each included sample, the mean and standard deviation of the outcomes were extracted separately for the intervention and control groups. Effect sizes were calculated as follows: for the denture base–tooth bonding studies, all of which reported shear bond strength, the mean difference (MD) and its 95% confidence interval were computed; for the liner–base bonding studies, which included both shear and tensile tests, the standardized mean difference (SMD) based on Hedges' g and its confidence interval was used. The pooled effect sizes were estimated using a random‐effects model based on the restricted maximum likelihood (REML) method (Deeks et al. 2024; Harville 1977). Heterogeneity among the included samples was evaluated using the *I* ^2^ statistic (Higgins and Thompson 2002), τ² statistic, and the *χ* ^2^ (*Q*) test (Cochran 1954). An *I* ^2^ > 50% and a *Q* test *p* < 0.05 were interpreted as indicators of substantial heterogeneity. To explore potential sources of heterogeneity, subgroup analyses were performed based on relevant moderator variables.

Publication bias was assessed using Egger's regression test and Begg's rank correlation test (Egger et al. 1997), with a *p* < 0.05 indicating potential bias. Additionally, funnel plots were generated for visual assessment, and the trim‐and‐fill method was applied to estimate the impact of any potentially missing studies on the overall effect size. A leave‐one‐out sensitivity analysis (Meng et al. 2024) was performed to evaluate the robustness of the pooled results by systematically excluding each study and observing its influence on the overall findings. All analyses were performed using R software version 4.3.1 (R Foundation for Statistical Computing) with the meta (version 6.5‐0) and metafor packages. Forest plots, funnel plots, and trim‐and‐fill analyses were generated using these packages, and all figures were produced directly in R.

### 2.6 Quality Assessment/Risk of Bias

Two reviewers (S.A. and A.M.) independently conducted a detailed quality evaluation of the included studies using the Quality Assessment Tool for In Vitro Studies (QUIN Tool) (Sheth et al. 2024). Each study received a risk of bias (RoB) score, which was classified as high (< 50%), moderate (50%–70%), or low (> 70%). Any disagreements between the reviewers were resolved by consulting a fifth reviewer (A.M.), ensuring consistency and accuracy in the quality assessment process.

### 2.7 Certainty of Evidence

The certainty of evidence for each outcome was assessed using the Grading of Recommendations, Assessment, Development, and Evaluation (GRADE) approach. Five domains were considered: risk of bias, inconsistency, indirectness, imprecision, and publication bias. Evidence was graded as high, moderate, low, or very low certainty.

## 3 Results

### 3.1 Study Selection

**Figure 1:**
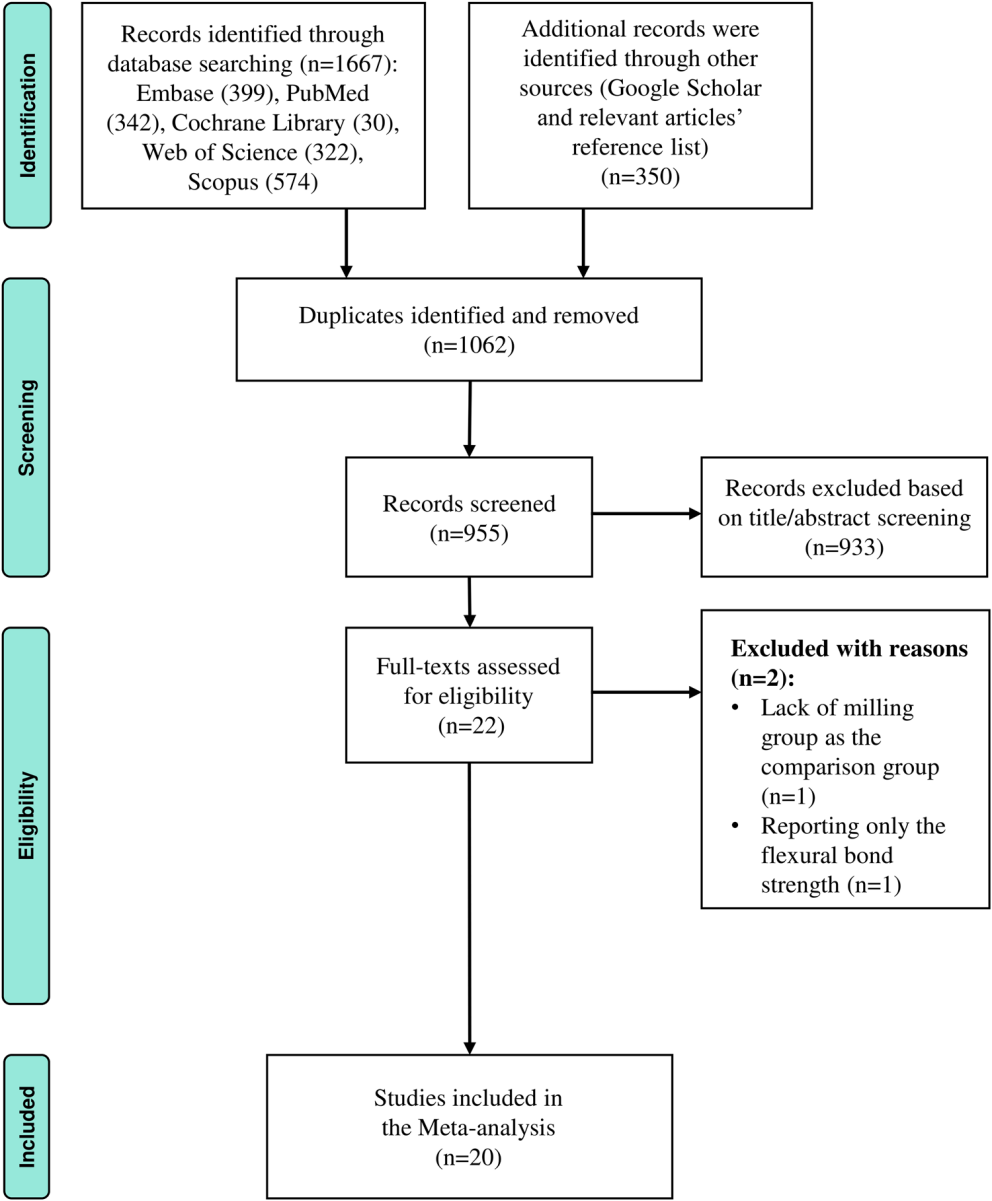
PRISMA flow diagram illustrating the identification, screening, exclusion, and inclusion process for eligible in vitro studies.

The initial search resulted in 2017 studies. During the title and abstract screening stage, duplicates (*n* = 1062) and irrelevant reports (% = 933) were excluded. After analyzing the full texts of the remaining 22 articles, two studies were excluded: one due to reporting only the flexural strength (Sahin et al. 2024a) and one lacked the milling group for comparison (Mohamed et al. 2023a) (Table 2). Ultimately, 20 articles were selected for the meta‐analysis (Figure 1) (Alanazi et al. 2024; Albazroun et al. 2024; Alfaraj et al. 2023; Awad et al. 2023; Choi et al. 2020; Gad et al. 2022; Gibreel et al. 2024; Htat et al. 2024; Janyaprasert et al. 2024; Kane and Shah 2023; Karaokutan and Ayvaz 2025; Karaokutan et al. 2024; Li et al. 2025; Löscher et al. 2024; Mert et al. 2023; A. Mohamed et al. 2023a; Sahin et al. 2024b; Tugut et al. 2024; Vuksic et al. 2023; Wemken et al. 2021).

**Table 2:** Overall and subgroup analyses of teeth bond strength.

	*N*	Pooled estimate		Heterogeneity			
		MD (95% CI)	*p* value	*I* ^2^	τ^2^	*p* value	*p* diff
Overall	41	5.317 (−9.792, 20.426)	0.490	100%	2300	< 0.001	NA
Overall^a^	39	−2.429 (−3.898, −0.960)	0.001	99.58%	15.55	< 0.001	NA
Study model							
Acrylic removable denture bases	8	9.762 (−7.121, 26.645)	0.257	33.54%	184.797	0.105	0.045
Digital model	25	−1.888 (−3.197, −0.578)	0.005	99.62%	11.039	< 0.001	
Intaglio surface of denture tooth	6	−25.646 (−47.900, −3.391)	0.024	99.82%	700.794	< 0.001	
Digitalization method							
Extraoral scanner	6	−25.646 (−47.900, −3.391)	0.024	99.82%	700.794	< 0.001	0.045
Intraoral scanner	8	9.762 (−7.121, 26.645)	0.257	33.54%	184.797	0.105	
NA	25	−1.888 (−3.197, −0.578)	0.005	99.62%	11.039	< 0.001	
Printer brand							
Cara Print 4.0, Kulzer	9	−0.773 (−0.951, −0.594)	< 0.001	72.99%	0.052	0.002	0.035
From 2, Formlabs	8	−7.361 (−27.199, 12.477)	0.467	59.30%	411.337	0.028	
From 3B+, Formlabs	16	−2.487 (−4.501, −0.474)	0.015	99.33%	16.702	< 0.001	
LulzBot TAZ6, Aleph Objects Inc.	2	21.756 (0.966, 42.546)	0.040	0.00%	0.000	0.354	
M2, Carbon	4	−27.834 (−60.506, 4.838)	0.095	99.93%	1063.196	< 0.001
DLP	9	−0.773 (−0.951, −0.594)	< 0.001	72.99%	0.052	0.002	0.015
DLS	4	−27.834 (−60.506, 4.838)	0.095	99.93%	1063.196	< 0.001	
FDM	2	21.756 (0.966, 42.546)	0.040	0.00%	0.000	0.354	
SLA	24	−2.659 (−4.670, −0.648)	0.010	99.01%	17.177	< 0.001	
Material (AM GROUP)							
Non‐PMMA resin	37	−2.543 (−4.017, −1.070)	0.001	99.61%	15.564	< 0.001	0.022
PMMA resin	2	21.756 (0.966, 42.546)	0.040	0.00%	0.000	0.354	
Material brand (AM GROUP)							
3D filament, Material4print	2	21.756 (0.966, 42.546)	0.040	0.00%	0.000	0.354	0.027
Denture Base Resin, Formlabs	22	−2.731 (−4.754, −0.708)	0.008	99.10%	17.240	< 0.001	
Dima Print Denture Base, Kulzer	9	−0.773 (−0.951, −0.594)	< 0.001	72.99%	0.052	0.002	
Grey Resin, Formlabs	2	5.791 (−19.612, 31.194)	0.655	33.77%	114.302	0.219	
Lucitone Digital Print, Dentsply Sirona	4	−27.834 (−60.506, 4.838)	0.095	99.93%	1063.196	< 0.001	
Layer thickness (µm)							
100	2	−55.427 (−84.357, −26.496)	< 0.001	76.65%	338.509	0.039	< 0.001
50	18	−2.738 (−4.761, −0.716)	0.008	99.26%	17.130	< 0.001	
NR	19	−0.831 (−1.025, −0.637)	< 0.001	63.00%	0.072	< 0.001	
Build orientation							
0	21	−2.490 (−4.488, −0.493)	0.015	99.11%	16.668	< 0.001	0.097
90	5	10.470 (−5.807, 26.748)	0.207	99.54%	216.290	0.028	
NR	13	−10.381 (−20.707, −0.055)	0.049	99.99%	332.844	< 0.001	
Specimen type (AM GROUP)							
Denture base	8	9.762 (−7.121, 26.645)	0.257	33.54%	184.797	0.105	0.153
Specimen	31	−2.586 (−4.067, −1.106)	0.001	99.67%	15.591	< 0.001	
Specimen dimension (AM GROUP)							
20 × 10 mm	2	−1.493 (−2.101, −0.885(	< 0.001	0.00%	0.000	0.345	0.010
25 × 4 × 3 mm	9	−0.773 (−0.951, −0.594(	< 0.001	72.99%	0.052	0.002	
64 × 10 × 3.3 mm	8	9.762 (−7.121, 26.645(	0.257	33.54%	184.797	0.105	
NR	20	−6.829 (−12.111, −1.546(	0.011	99.89%	132.118	< 0.001	
Specimen shape (AM GROUP)							
Beam	13	−10.381 (−20.707, −0.055)	0.049	99.99%	332.844	< 0.001	0.199
Cylindrical	2	−1.493 (−2.101, −0.885)	< 0.001	0.00%	0.000	0.345	
Denture shape	24	−2.199 (−4.212, −0.186)	0.032	99.00%	17.087	< 0.001	
Milling machine brand							
CORiTEC 350 Pro+, imes‐icore	8	−0.642 (−2.573, 1.290)	0.515	98.07%	7.569	< 0.001	0.004
NR	11	−0.834 (−1.029, −0.639)	< 0.001	75.56%	0.073	< 0.001	
PrograMill PM7, Ivoclar	8	−4.342 (−7.477, −1.207)	0.007	99.53%	20.298	< 0.001	
Roland DWX‐50, Roland DG Corporation	8	9.762 (−7.121, 26.645)	0.257	33.54%	184.797	0.105	
VersaMil Inc	4	−39.477 (−64.903, −14.051)	0.002	84.65%	558.964	< 0.001	
Number of milling axis							
5	28	−5.232 (−10.498, 0.035)	0.052	99.86%	147.735	< 0.001	0.102
NR	11	−0.834 (−1.029, −0.639)	< 0.001	75.56%	0.073	< 0.001	
Material (SM GROUP)							
PMMA resin	39	−2.429 (−3.898, −0.960)	0.001	99.58%	15.549	< 0.001	NA
Material brand (SM GROUP)							
IvoBase CAD, Ivoclar Vivodent	19	−0.779 (−0.959, −0.598)	< 0.001	55.32%	0.054	< 0.001	< 0.001
Ivotion Base Disc, Ivoclar Vivodent	4	−1.004 (−3.911, 1.904)	0.499	99.02%	8.657	< 0.001	
Ivotion Disc, Ivoclar Vivodent	4	−7.714 (−10.621, −4.806)	< 0.001	98.37%	8.617	< 0.001	
Polident PMMA, Polident Dental	2	−55.521 (−88.691, −22.350)	0.001	70.46%	403.939	0.066	
Vionic Base Disc, Vita Zahnfabrik	8	−0.642 (−2.573, 1.290)	0.515	98.07%	7.569	< 0.001	
XCL1; AvaDent, AvaDent Digital Dental Solutions	2	−1.493 (−2.101, −0.885)	< 0.001	0.00%	0.000	0.345	
Specimen type (SM GROUP)							
Denture base	8	9.762 (−7.121, 26.645)	0.257	33.54%	184.797	0.105	0.153
Specimen	31	−2.586 (−4.067, −1.106)	0.001	99.67%	15.591	< 0.001	
Specimen dimension (SM GROUP)							
20 × 10 mm	2	−1.493 (−2.101, −0.885)	< 0.001	0.00%	0.000	0.345	0.010
25 × 4 × 3 mm	9	−0.773 (−0.951, −0.594)	< 0.001	72.99%	0.052	0.002	
64 × 10 × 3.3 mm	8	9.762 (−7.121, 26.645)	0.257	33.54%	184.797	0.105	
NR	20	−6.829 (−12.111, −1.546)	0.011	99.89%	132.118	< 0.001	
Specimen shape (SM GROUP)							
Beam	13	−10.381 (−20.707, −0.055)	0.049	99.99%	332.844	< 0.001	0.199
Cylindrical	2	−1.493 (−2.101, −0.885)	< 0.001	0.00%	0.000	0.345	
Denture shape	24	−2.199 (−4.212, −0.186)	0.032	99.00%	17.087	< 0.001	
Bonded teeth material (AM GROUP)							
NR	8	9.762 (−7.121, 26.645)	0.257	33.54%	184.797	0.105	0.049
Non‐PMMA resin	17	−1.243 (−2.803, 0.316)	0.118	99.71%	10.661	< 0.001	
PMMA‐based resin	14	−10.868 (−19.788, −1.947)	0.017	99.92%	265.422	< 0.001	
Chemical composition of bonded teeth (AM GROUP)							
Interpenetrating polymer network	2	−1.493 (−2.101, −0.885)	< 0.001	0.00%	0.000	0.345	< 0.001
Microfiller reinforced polymer matrix composite	4	−39.477 (−64.903, −14.051)	0.002	84.65%	558.964	< 0.001	
Multiconstituent substance	9	−0.773 (−0.951, −0.594)	< 0.001	72.99%	0.052	0.002	
NR	10	2.273 (−1.101, 5.647)	0.187	92.91%	12.192	< 0.001	
Nano‐ceramic filled biocompatible material	4	−5.155 (−8.584, −1.725)	0.003	99.53%	12.185	< 0.001	
Plastic teeth	2	10.988 (−51.911, 73.887)	0.732	79.22%	1634.087	0.028	
SE polymer composite	8	−3.268 (−5.518, −1.019)	0.004	98.67%	10.357	< 0.001	
Bonded teeth brand (AM GROUP)							
Denture Base Resin, Formlabs	4	1.795 (−1.634, 5.225)	0.305	97.46%	11.937	< 0.001	< 0.001
Denture Teeth Resin, Formlabs	4	−5.155 (−8.584, −1.725)	0.003	99.53%	12.185	< 0.001	
Dima Print Denture Teeth, Kulzer	9	−0.773 (−0.951, −0.594)	< 0.001	72.99%	0.052	0.002	
IPN 3D print tooth material, Dentsply Sirona	2	−1.493 (−2.101, −0.885)	< 0.001	0.00%	0.000	0.345	
NR	8	9.762 (−7.121, 26.645)	0.257	33.54%	184.797	0.105	
Vionic Dent Disc, Vita Zahnfabrik	Pooled effect size estimation was not feasible in this subgroup due to missing standard deviation data for the intervention group						
Vionic Vigo, Vita Zahnfabrik	8	−3.268 (−5.518, −1.019)	0.004	98.67%	10.357	< 0.001	
Vita Vitapan, Vita Zahnfabrik	4	−39.477 (−64.903, −14.051)	0.002	84.65%	558.964	< 0.001	
Bonded teeth shape							
Beam	9	−0.773 (−0.951, −0.594)	< 0.001	72.99%	0.052	0.002	0.033
NR	8	9.762 (−7.121, 26.645)	0.257	33.54%	184.797	0.105	
Tooth shape	22	−5.717 (−9.908, −1.526)	0.008	99.84%	90.614	< 0.001	
Bonding agent (AM GROUP)							
Denture Base Resin, Formlabs	8	−4.121 (−6.494, −1.749)	0.001	99.42%	11.644	< 0.001	< 0.001
Dima Print Denture Base, Kulzer	9	−0.773 (−0.951, −0.594)	< 0.001	72.99%	0.052	0.002	
Ivobase CAD bonding system, Ivoclar	4	−39.477 (−64.903, −14.051)	0.002	84.65%	558.964	< 0.001	
Lucitone digital fuse step 2, Dentsply Sirona	2	−1.493 (−2.101, −0.885)	< 0.001	0.00%	0.000	0.345	
NA	4	1.795 (−1.634, 5.225)	0.305	97.46%	11.937	< 0.001	
NR	6	12.820 (−4.192, 29.833)	0.140	39.09%	173.865	0.126	
Self‐cure resin (ortho resin)	1	−61.360 (−181.152, 58.432)	0.315	NA	0.000	NA	
Uncured denture base resin	1	−68.870 (−183.124, 45.384)	0.237	NA	0.000	NA	
Vionic Bond, Vita Zahnfabrik	4	−3.455 (−6.884, −0.025)	0.048	97.82%	11.980	< 0.001	
Teeth pretreatment (AM GROUP)							
Mechanical and chemical treatment	2	−65.293 (−147.971, 17.386)	0.122	0.00%	0.000	0.929	0.009
Mechanical treatment	16	−9.971 (−17.058, −2.883)	0.006	99.93%	190.024	< 0.001	
NA	4	1.795 (−1.634, 5.225)	0.305	97.46%	11.937	< 0.001	
NR	11	−0.834 (−1.029, −0.639)	< 0.001	75.56%	0.073	< 0.001	
No treatment	6	12.820 (−4.192, 29.833)	0.140	39.09%	173.865	0.126	
Bonded teeth material (SM GROUP)							0.153
NR	8	9.762 (−7.121, 26.645)	0.257	33.54%	184.797	0.105	
PMMA‐based resin	31	−2.586 (−4.067, −1.106)	0.001	99.67%	15.591	< 0.001	
Chemical composition of bonded teeth (SM GROUP)							
Double Cross‐linked PMMA	3	−0.732 (−0.876, −0.589)	< 0.001	0.00%	0.000	0.536	0.002
Extreme cross‐linked PMMA	2	−1.493 (−2.101, −0.885)	< 0.001	0.00%	0.000	0.345	
Microfiller reinforced polymer matrix composite	8	−18.614 (−36.264, −0.963)	0.039	99.94%	596.478	< 0.001	
Monochromatic dental ceramic layer (DCL) material	4	−1.004 (−3.911, 1.904)	0.499	99.02%	8.657	< 0.001	
NR	12	−6.649 (−9.855, −3.443)	< 0.001	95.55%	11.195	< 0.001	
PMMA with nano fillers	3	−0.897 (−1.574, −0.221)	0.009	93.75%	0.325	< 0.001	
SE polymer composite	4	−0.184 (−3.091, 2.724)	0.901	98.24%	8.607	< 0.001	
Unfilled PMMA	3	−0.803 (−1.155, −0.451)	< 0.001	74.57%	0.072	0.027	
Bonded teeth brand (SM GROUP)							
Ivoclar DCL, Ivoclar Vivodent	3	−0.732 (−0.876, −0.589)	< 0.001	0.00%	0.000	0.536	< 0.001
Ivoclar SPE, Ivoclar Vivodent	3	−0.803 (−1.155, −0.4510	< 0.001	74.57%	0.072	0.027	
Ivotion Dent Disc, Ivoclar Vivodent	4	−1.004 (−3.911, 1.904)	0.499	99.02%	8.657	< 0.001	
Ivotion Disc, Ivoclar Vivodent	4	−7.714 (−10.621, −4.806)	< 0.001	98.37%	8.617	< 0.001	
Mondial, Kulzer	3	−0.897 (−1.574, −0.221)	0.009	93.75%	0.325	< 0.001	
NR	8	9.762 (−7.121, 26.645)	0.257	33.54%	184.797	0.105	
Vionic Dent Disc, Vita Zahnfabrik	4	−1.094 (−4.001, 1.814)	0.461	98.12%	8.599	< 0.001	
Vionic Vigo, Vita Zahnfabrik	4	−0.184 (−3.091, 2.724)	0.901	98.24%	8.607	< 0.001	
Vita Vitapan, Vita Zahnfabrik	4	−39.477 (−64.903, −14.051)	0.002	84.65%	558.964	< 0.001	
XCL1; AvaDent, Digital Dental Solutions	2	−1.493 (−2.101, −0.885)	< 0.001	0.00%	0.000	0.345	
Bonding agent (SM GROUP)							
Ivobase CAD bond, Ivoclar Vivodent	13	−10.381 (−20.707, −0.055)	0.049	99.99%	332.844	< 0.001	0.017
Ivotion Bond, Ivoclar Vivodent	4	−1.004 (−3.911, 1.904)	0.499	99.02%	8.657	< 0.001	
NA	6	−5.692 (−8.808, −2.577)	< 0.001	98.98%	14.954	< 0.001	
NR	8	9.762 (−7.121, 26.645)	0.257	33.54%	184.797	0.105	
Vionic Bond, Vita Zahnfabrik	8	−0.642 (−2.573, 1.290)	0.515	98.07%	7.569	< 0.001	
Teeth pretreatment (SM GROUP)							
Mechanical and chemical treatment	2	−65.293 (−147.971, 17.386)	0.122	0.00%	0.000	0.929	0.002
Mechanical treatment	16	−7.821 (−15.726, 0.085)	0.053	99.94%	239.619	< 0.001	
NA	6	−5.692 (−8.808, −2.577)	< 0.001	98.98%	14.954	< 0.001	
NR	9	−0.773 (−0.951, −0.594)	< 0.001	72.99%	0.052	0.002	
No treatment	6	12.820 (−4.192, 29.833)	0.140	39.09%	173.865	0.126	
Aging process							
Distilled water	4	−39.477 (−64.903, −14.051)	0.002	84.65%	558.964	< 0.001	0.009
NA	8	9.762 (−7.121, 26.645)	0.257	33.54%	184.797	0.105	
No aging	4	−1.097 (−1.732, −0.462)	0.001	88.72%	0.333	0.001	
Thermocycling	23	−1.983 (−3.403, −0.563)	0.006	99.61%	11.929	< 0.001	
Aging duration (month)							
12	4	−0.922 (−1.192, −0.652)	< 0.001	63.64%	0.044	0.056	0.001
24	4	−39.477 (−64.903, −14.051)	0.002	84.65%	558.964	< 0.001	
6	3	−0.594 (−0.738, −0.450)	< 0.001	0.00%	0.000	0.448	
NA	12	−1.069 (−1.698, −0.439)	0.001	67.80%	0.327	0.001	
NR	16	−2.487 (−4.501, −0.474)	0.015	99.33%	16.702	< 0.001	
Thermocycling cycles							
10,000	1	−1.320 (−2.026, −0.614)	< 0.001	NA	0.000	NA	0.031
1200	3	−0.872 (−1.147, −0.596)	< 0.001	68.33%	0.040	0.055	
600	3	−0.594 (−0.738, −0.450)	< 0.001	0.00%	0.000	0.448	
6000	16	−2.487 (−4.501, −0.474)	0.015	99.33%	16.702	< 0.001	
NA	16	−8.649 (−23.036, 5.738)	0.239	99.97%	636.350	< 0.001	

Abbreviations: AM GROUP, additive manufacturing group; CI, confidence interval; DLP, digital light processing; DLS, digital light synthesis; FDM, fused deposition modeling; I², percentage of total variation due to heterogeneity; MD, mean difference; N, number of included comparisons; NA, not applicable; NR, not reported; *p* diff, *p* value for difference between subgroups; *p* (heterogeneity), *p* value assessing heterogeneity; PMMA, polymethyl methacrylate; *p* value, probability value for statistical significance of MD; SLA, stereolithography; SM GROUP, subtractive manufacturing group; τ², between‐study variance in random‐effects meta‐analysis.

^a^ After exclusion of sensitivity‐influencing studies (Alanazi and colleagues #7 and #8).

### 3.2 Study Characteristics

The selected studies were published between 2020 and 2024. Four studies were conducted in the United States (Alfaraj et al. 2023; Awad et al. 2023; Kane and Shah 2023; Li et al. 2025), four in Türkiye (Karaokutan and Ayvaz 2025; Karaokutan et al. 2024; Sahin et al. 2024b; Tugut et al. 2024), two in Saudi Arabia (Albazroun et al. 2024; Gad et al. 2022), two in Thailand (Htat et al. 2024; Janyaprasert et al. 2024), two in Germany (Löscher et al. 2024; Wemken et al. 2021), and one each in New Zealand (Choi et al. 2020), Japan (A. Mohamed et al. 2023a), the UK (Alanazi et al. 2024), Croatia (Vuksic et al. 2023), Finland (Gibreel et al. 2024), and Switzerland (Mert et al. 2023).

Regarding the tested paramteters, five studies investigated the denture teeth bond strength (Alanazi et al. 2024; Choi et al. 2020; Kane and Shah 2023; Löscher et al. 2024; A. Mohamed et al. 2023a), and 15 studies the liner bond strength to the denture base (Albazroun et al. 2024; Alfaraj et al. 2023; Awad et al. 2023; Gad et al. 2022; Gibreel et al. 2024; Htat et al. 2024; Janyaprasert et al. 2024; Karaokutan and Ayvaz 2025; Karaokutan et al. 2024; Li et al. 2025; Mert et al. 2023; Sahin et al. 2024b; Tugut et al. 2024; Vuksic et al. 2023; Wemken et al. 2021). All studies used a digital model, while two used the intaglio surface of denture tooth (Kane and Shah 2023; A. Mohamed et al. 2023a), and one focused on acrylic removable denture bases (Alanazi et al. 2024).

Information on the digitalization method was not available for most studies, except for three that utilized intraoral (Alanazi et al. 2024) (brand was not reported) and extraoral (D750, 3Shape, New Providence, NJ (Kane and Shah 2023), EDGE 3D Model Scanner, DOF Inc, Seoul, Korea (A. Mohamed et al. 2023a)) scanners. Various design software was employed across the studies, including Fusion 360 (Autodesk, Mill Valley, California, US) (Alanazi et al. 2024; Gibreel et al. 2024; Wemken et al. 2021), Meshmixer (Autodesk Inc, San Rafael, CA) (Awad et al. 2023; Kane and Shah 2023; Karaokutan and Ayvaz 2025; Karaokutan et al. 2024; Li et al. 2025; A. Mohamed et al. 2023a), Dassault Systèmes (SolidWorks Corp., Waltham, USA) (Janyaprasert et al. 2024; Sahin et al. 2024b; Tugut et al. 2024), 3Shape Dental System DS (3Shape, Copenhagen, Denmark) (Htat et al. 2024), AutoCAD (Autodesk, USA) (Tugut et al. 2024); Additionally, six studies did not specify the software used.

Regarding 3D printing, the technologies used included DLP (Albazroun et al. 2024; Choi et al. 2020; Gad et al. 2022; Gibreel et al. 2024; Htat et al. 2024; Janyaprasert et al. 2024; Karaokutan and Ayvaz 2025; Karaokutan et al. 2024; Li et al. 2025; Tugut et al. 2024; Vuksic et al. 2023; Wemken et al. 2021), SLA (Alanazi et al. 2024; Albazroun et al. 2024; Awad et al. 2023; Gad et al. 2022; Kane and Shah 2023; Li et al. 2025; Löscher et al. 2024), DLS (Alfaraj et al. 2023; Kane and Shah 2023; Li et al. 2025; A. Mohamed et al. 2023a), LCD (Sahin et al. 2024b), and FDM (Alanazi et al. 2024). Furthermore, one study did not indicate the printing technology used (Mert et al. 2023).

The 3D printers represented were LulzBot TAZ6 (Aleph Objects Inc., Colorado, USA) (Alanazi et al. 2024), Form 2 (Formlabs Inc., Somerville, MA, USA) (Alanazi et al. 2024; Albazroun et al. 2024; Gad et al. 2022; Kane and Shah 2023), Asiga Max (Asiga, SCHEU‐DENTAL GmbH, Germany) (Gad et al. 2022; Gibreel et al. 2024), M2 (Carbon Inc, Redwood City, CA, USA) (Alfaraj et al. 2023; Kane and Shah 2023; Li et al. 2025; A. Mohamed et al. 2023a), SolFlex 170 (VOCO, Cuxhaven, Germany) (Wemken et al. 2021), SolFlex 650 (VOCO, Cuxhaven, Germany) (Karaokutan and Ayvaz 2025; Karaokutan et al. 2024), NextDent 5100 (NextDent, Soesterberg, Netherlands) (Albazroun et al. 2024; Gad et al. 2022; Janyaprasert et al. 2024), Form 3B+ (Formlabs, USA) (Li et al. 2025), FreeShape 120 (Ackuretta, Neukiritzsch, Germany) (Sahin et al. 2024b), CARES P30 (Straumman, Basel, Switzerland) (Li et al. 2025), Ackuretta (Tugut et al. 2024), while three studies did not report the printer used (Awad et al. 2023; Mert et al. 2023; Vuksic et al. 2023).

All printing materials were non‐PMMA resins, except for two studies that also used PMMA resins (Alanazi et al. 2024; Vuksic et al. 2023). The PMMA resin brands were 3D Filament (Material4print, Germany) (Alanazi et al. 2024) and Imprimo LC denture (Scheu, Iserlohn, Germany) (Vuksic et al. 2023), while the non‐PMMA resins included Grey Resin (Formlabs, USA) (Alanazi et al. 2024), Denture Base Resin (Formlabs, USA) (Kane and Shah 2023; Löscher et al. 2024), Denture Base LP Resin (Formlabs, USA) (Awad et al. 2023; Gad et al. 2022), Denture 3D+ (NextDent, Soesterberg, Netherlands) (Albazroun et al. 2024; Gad et al. 2022; Mert et al. 2023), Dentca (DENTCA, California, USA) (Li et al. 2025), Lucitone Digital Print (Dentsply Sirona, Charlotte, NC, USA) (Alfaraj et al. 2023; Kane and Shah 2023; Li et al. 2025; A. Mohamed et al. 2023a), MACK4D Denture Light Pink (Dentona, Germany) (Tugut et al. 2024), Curo Denture, MACK4D (Ackuretta, Neukiritzsch, Germany) (Sahin et al. 2024b), FreePrint Denture (Detax, Ettlingen, Germany) (Gibreel et al. 2024; Vuksic et al. 2023), Dima Print Denture Base (Kulzer, USA) (Choi et al. 2020), FotoDent (Dreve Dentamid, Unna, Germany) (Li et al. 2025), DentaBASE (ASIGA, Germany) (Gad et al. 2022), and V‐Print dentbase (VOCO, Cuxhaven, Germany) (Gibreel et al. 2024; Karaokutan and Ayvaz 2025; Karaokutan et al. 2024).

The milling machines reported were primarily 5‐axis, with eight studies not specifying the type (Albazroun et al. 2024; Awad et al. 2023; Choi et al. 2020; Gad et al. 2022; Gibreel et al. 2024; Mert et al. 2023; Tugut et al. 2024; Vuksic et al. 2023). All materials utilized for milling were PMMA resins from various brands, including AvaDent (AvaDent Digital Dental Solutions, Scottsdale, AZ, USA) (Albazroun et al. 2024; Gad et al. 2022; A. Mohamed et al. 2023b), IvoBase CAD (Ivoclar Vivadent, Amherst, NY, USA) (Alanazi et al. 2024; Albazroun et al. 2024; Alfaraj et al. 2023; Awad et al. 2023; Choi et al. 2020; Gad et al. 2022; Kane and Shah 2023; Li et al. 2025; Mert et al. 2023; Vuksic et al. 2023; Wemken et al. 2021), Polident (Polident, Volˇcja draga, Slovenia) (Kane and Shah 2023; Karaokutan and Ayvaz 2025; Karaokutan et al. 2024; Vuksic et al. 2023), Anaxdent (Anaxdent, Stuttgart, Germany) (Vuksic et al. 2023), L‐Temp (Degos Dental, Bayern, Germany) (Gibreel et al. 2024), Temp Basic Tissue (Zirkonzahn, Gais, Italy) (Gibreel et al. 2024), Ivotion Base Disc (Ivoclar Vivadent, Schaan, Liechtenstein) (Löscher et al. 2024; Mert et al. 2023), Vionic Base Disc (Vita zahnfabrik, Germany) (Löscher et al. 2024), Yamahachi (Yamahachi Dental MFG, Aichi‐Pref, Japan) (Sahin et al. 2024b; Tugut et al. 2024), Smile Cam (Pressing Dental, San Marino, Italy) (Janyaprasert et al. 2024) and Luciton 199 Denture Base Disc (Dentsply Sirona, Charlotte, NC, USA) (Li et al. 2025).

The characteristics of the included studies are detailed in Supporting Tables 2 and 3. In some cases, multiple intervention comparison pairs were extracted from a single study, each treated as a separate data point in the meta‐analysis.

### 3.3 Meta‐Analyses

#### 3.3.1 Tooth Bond Strength

**Figure 2:**
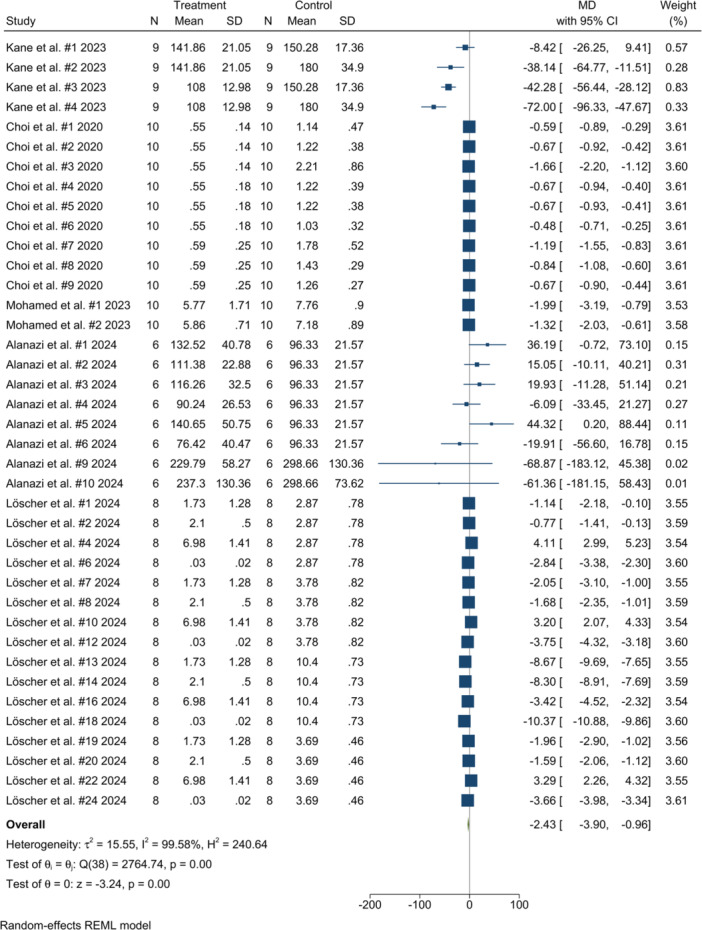
Forest plot of overall tooth–base bond strength comparisons between AM and SM denture bases after removal of two outlier comparisons.

**Figure 3:**
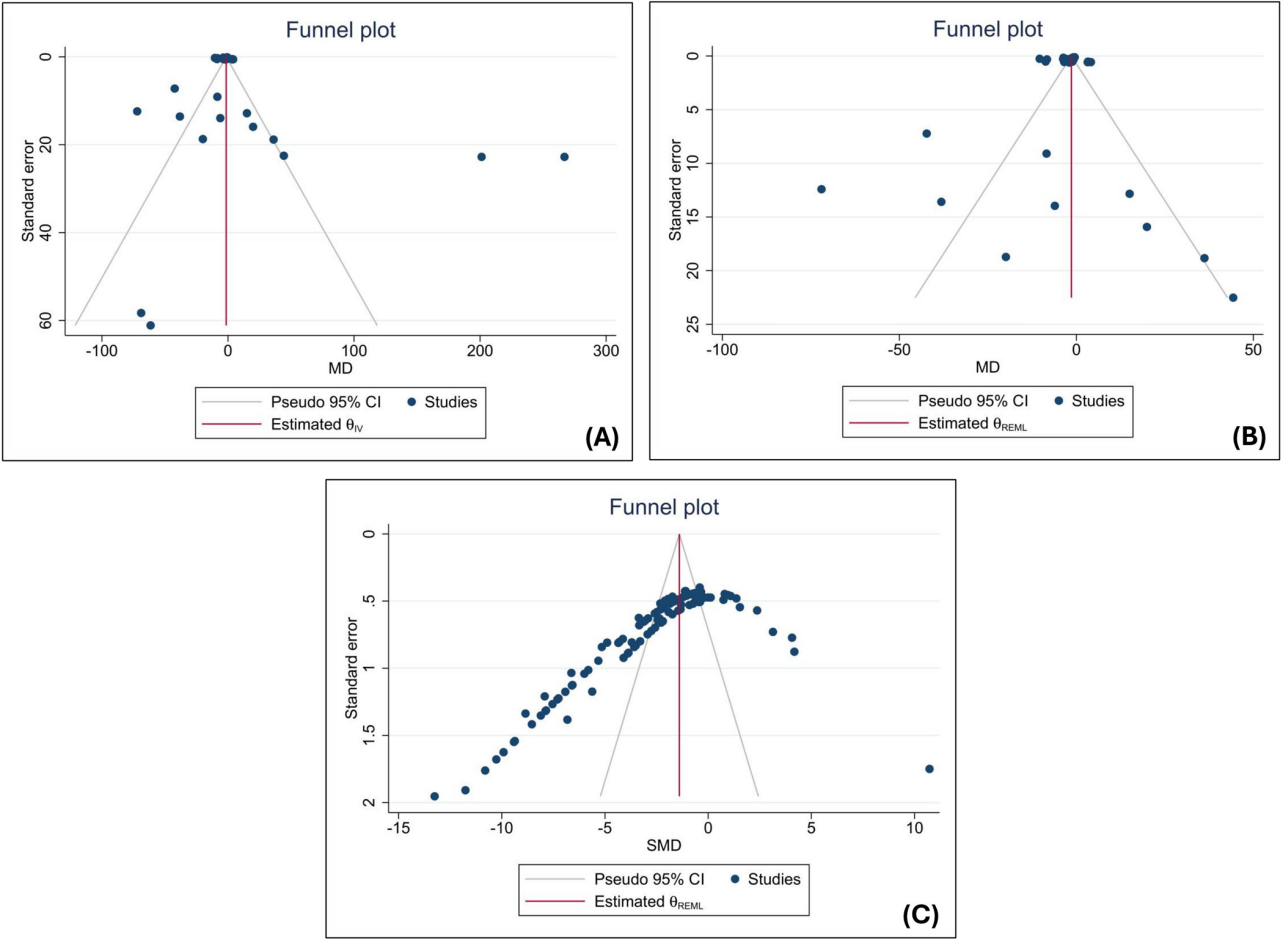
Funnel plots assessing publication bias in (A) all tooth–base bond strength studies, (B) tooth–base bond strength studies after removal of the two outliers, and (C) reline–base bond strength studies.

The initial random‐effects synthesis of all 41 comparisons showed no statistically significant difference in mean shear bond strength between teeth bonded to AM and SM denture bases (MD = 5.32 MPa, 95% CI–9.79–20.43; *p *= 0.49; τ^2^ = 2300; *I* ^2^ = 100%). Leave‐one‐out analysis confirmed that omitting any single comparison did not resolve the extreme heterogeneity, although two outlying comparisons from Alanazi and colleagues (#7 and #8) were consistently flagged by influence diagnostics (Figure S1). After excluding these two sensitivity‐driving studies (39 comparisons), between‐study variance fell sharply (τ^2^ = 15.55 MPa^2^) and the pooled effect favored milled bases (MD = −2.43 MPa, 95% CI–3.90 to −0.96; *p *= 0.001), indicating that tooth‐to‐base bond strength was significantly lower for AM compared with SM bases (Figure 2). Renewed leave‐one‐out analysis demonstrated stable, consistently negative pooled estimates (range −2.04 to −2.61 MPa) whose confidence intervals no longer crossed zero, indicating a robust finding (Figure S2). Publication‐bias diagnostics pointed to persistent small‐study effects. Publication‐bias diagnostics remained significant both before (Egger β_1_ = 5.01 ± 0.59, *z* = 8.51; Begg *z* = 4.43; both *p* < 0.001) and after (β_1_ = 4.53 ± 0.45, *z* = 10.09; Begg *z* = 3.99, *p *< 0.001) exclusion of the two influential comparisons and the corresponding funnel plots (Figure 3A,B) showed persistent right‐sided asymmetry, indicating small‐study effects. The trim‐and‐fill procedure did not identify or impute any missing studies.

Within the AM strata, every statistically significant moderator—except one—favored the subtractive group. Bonds were significantly weaker when printed with DLP or SLA technologies, when 50 or 100 µm layers were deposited, and when the build angle was 0 degrees. The deficit also appeared with non‐PMMA photopolymers, with beam, cylindrical, or denture‐shaped test pieces, and whenever the test involved isolated specimens rather than whole denture bases. Joining PMMA‐printed bases to PMMA teeth—especially those formulated as interpenetrating‐polymer‐network, micro‐filler‐reinforced, nano‐filled, or multiconstituent composites—further reduced adhesion, as did using methacrylate‐monomer primers or no bonding agent and applying mechanical surface roughening to the tooth intaglio. The sole subgroup that reversed the trend was fused‐deposition‐modeled PMMA filament (Material4Print), which yielded a significantly stronger joint than its milled counterpart (MD ≈ + 21.8 MPa). However, within the SM cohort, several conditions produced even higher bond strengths. Significant gains were recorded for milled PMMA discs, for beam, denture‐shaped, and plate/cylinder specimens, and for bonds to PMMA teeth, particularly those that were double‐ or extreme‐cross‐linked, micro‐filler‐reinforced, nano‐filled, or unfilled. Adhesion also improved when the joint was made with the proprietary Ivobase CAD Bond system or when no dedicated adhesive was used, and it remained superior after either minimal mechanical surface preparation or when pretreatment was simply not reported.

#### 3.3.2 Reline Bond Strength

**Figure 4:**
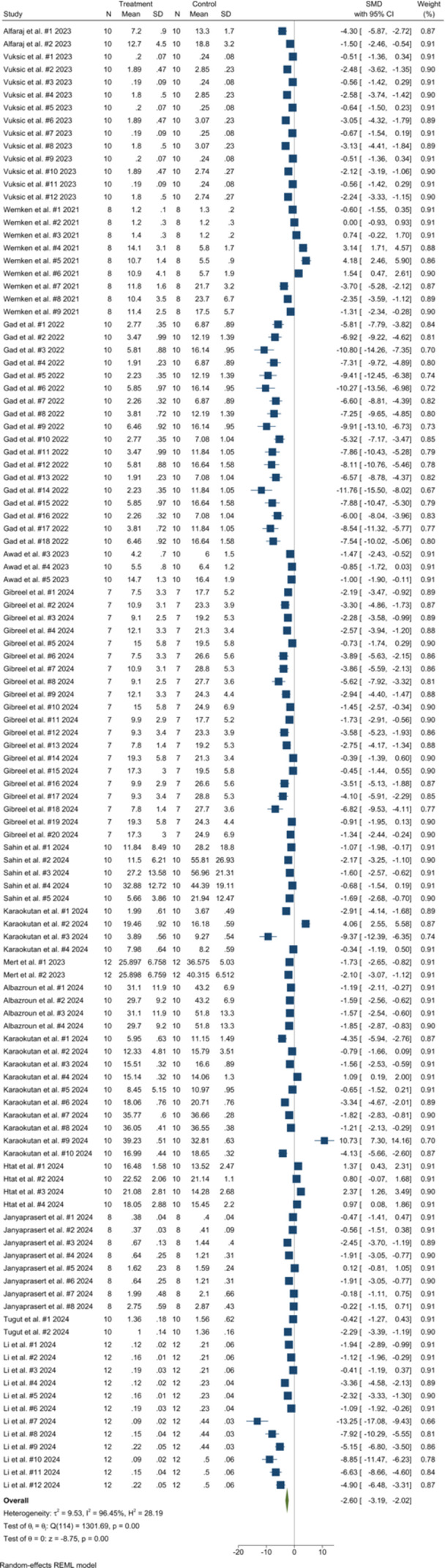
Forest plot of overall reline–base bond strength comparisons between AM and SM denture bases.

The random‐effects synthesis of all 115 comparisons (Figure 4) demonstrated a large and highly significant reduction in reline‐to‐base bond strength for AM denture bases compared with SM ones (pooled SMD = –2.603, 95% CI –3.186 to –2.020; *p* < 0.001; τ^2^ = 9.53; *I* ^2^ = 96.5%), meaning that AM bases consistently showed lower adhesion values than SM bases. Leave‐one‐out sensitivity analysis shifted the pooled estimate by no more than ±0.07 SMD units (range –2.53 to –2.68) and left both τ^2^ and *I* ^2^ essentially unchanged, confirming that no single study materially influenced the overall finding. Publication‐bias diagnostics indicated pronounced small‐study effects: Egger's intercept β_1_ = –6.98 ± 0.53 (*z* = –13.14, *p* < 0.001) and Begg's rank‐correlation test *z *= –10.69 (*p *< 0.001); the funnel plot revealed marked left‐side asymmetry (Figure 3C). Trim‐and‐fill analyses did not impute any missing studies.

Subgroup analysis results (Table 3) demonstrated that Printing with DLP, DLS, LCD, or SLA technologies, using 50 or 100 µm layers, and building at 0° orientation all yielded significantly weaker bonds for printed bases. The disadvantage also appeared with both non‐PMMA and PMMA photo‐resins and when specimens were configured as beams or plate/cylindrical blocks. Among liner‐related variables, bonds were lower when the liner was acrylate‐based or silicone‐based, when it was hard or soft, and when it was auto‐polymerized or heat‐polymerized. Additional moderators that reduced AM adhesion included the use of methacrylate‐monomer primers or no bonding agent and any form of chemical, mechanical, combined, physicochemical, or absent surface pretreatment of the denture resin. SM‐group conditions were associated with even stronger SM performance. Within the subtractive strata, statistically higher bond strengths persisted for PMMA discs, for beam and plate/cylindrical specimens, and whenever relining employed acrylate‐ or silicone‐based liners, regardless of whether they were hard or soft or auto‐ or heat‐polymerized. The SM advantage was also maintained when methacrylate monomer or no bonding agent was used, and after mechanical surface preparation or no pretreatment of the base resin.

**Table 3:** Overall and subgroup analyses of liners.

	*N*	Pooled estimate		Heterogeneity			
		SMD (95% CI)	*p* value	I^2^	τ^2^	*p* value	*p* diff
Overall	115	−2.603 (−3.186, −2.020)	< 0.001	96.45%	9.534	< 0.001	NA
Printer brand							
Ackuretta	2	−1.323 (−3.158, 0.513)	0.158	85.68%	1.504	0.008	< 0.001
Asiga Max, Asiga	26	−3.570 (−4.504, −2.636)	< 0.001	90.34%	5.115	< 0.001	
CARES P30, Straumman	4	−6.613 (−11.601, −1.626)	0.009	97.26%	24.419	< 0.001	
Form 2, Formlabs	8	−5.939 (−8.086, −3.792)	< 0.001	91.31%	8.332	< 0.001	
FreeShape 120, Ackuretta	5	−1.387 (−1.894, −0.880)	< 0.001	28.86%	0.097	0.231	
M2, Carbon	10	−3.382 (−4.946, −1.817)	< 0.001	94.58%	5.839	< 0.001	
NR	17	−1.464 (−1.894, −1.033)	< 0.001	70.23%	0.567	< 0.001	
NextDent 5100, NextDent	20	−2.677 (−4.446, −0.908)	0.003	97.83%	15.504	< 0.001	
SolFlex 170, VOCO	9	0.164 (−1.437, 1.765)	0.841	94.31%	5.608	< 0.001	
SolFlex 650, VOCO	14	−1.090 (−3.277, 1.097)	0.329	98.04%	16.776	< 0.001	
Printing technology							
DLP	87	−2.367 (−3.066, −1.667)	< 0.001	96.65%	10.415	< 0.001	0.004
DLS	6	−3.806 (−5.997, −1.616)	0.001	94.56%	6.877	< 0.001	
LCD	5	−1.387 (−1.894, −0.880)	< 0.001	28.86%	0.097	0.231	
NR	2	−1.903 (−2.569, −1.236)	< 0.001	0.00%	0.000	0.591	
SLA	15	−4.055 (−5.649, −2.462)	< 0.001	95.92%	9.102	< 0.001	
Material (AM GROUP)							
Non‐PMMA resin	109	−2.668 (−3.284, −2.051)	< 0.001	96.57%	10.109	< 0.001	0.047
PMMA‐based resin	6	−1.553 (−2.462, −0.643)	0.001	80.05%	1.022	< 0.001	
Material brand (AM GROUP)							
Curo Denture, MACK4D, Ackuretta	5	−1.387 (−1.894, −0.880)	< 0.001	28.86%	0.097	0.231	< 0.001
DentaBASE, ASIGA	6	−7.107 (−8.484, −5.730)	< 0.001	50.03%	1.449	0.070	
Dentca, DENTCA	4	−4.356 (−7.560, −1.151)	0.008	95.68%	9.975	< 0.001	
Denture 3D+, NextDent	22	−2.581 (−4.170, −0.992)	0.001	97.66%	13.724	< 0.001	
Denture Base LP Resin, Formlabs	11	−4.561 (−6.569, −2.553)	< 0.001	95.84%	10.576	< 0.001	
FotoDent, Dreve Dentamid	4	−6.613 (−11.601, −1.626)	0.009	97.26%	24.419	< 0.001	
Freeprint denture, Detax	16	−1.958 (−2.681, −1.236)	< 0.001	83.80%	1.742	< 0.001	
Imprimo LC denture, Scheu	6	−1.553 (−2.462, −0.643)	0.001	80.05%	1.022	< 0.001	
Lucitone Digital Print, Dentsply Sirona	6	−2.787 (−4.481, −1.093)	0.001	92.82%	4.063	< 0.001	
MACK4D Denture Light Pink, Dentona	2	−1.323 (−3.158, 0.513)	0.158	85.68%	1.504	0.008	
V‐Print dentbase, VOCO	33	−1.281 (−2.338, −0.224)	0.018	96.08%	9.047	< 0.001	
Layer thickness (µm)							
100	2	−1.323 (−3.158, 0.513)	0.158	85.68%	1.504	0.008	0.104
50	60	−3.209 (−4.093, −2.326)	< 0.001	96.26%	11.368	< 0.001	
65	14	−1.090 (−3.277, 1.097)	0.329	98.04%	16.776	< 0.001	
NR	39	−2.230 (−2.903, −1.556)	< 0.001	93.75%	4.189	< 0.001	
Build orientation							
0	41	−3.739 (−4.932, −2.546)	< 0.001	97.12%	14.259	< 0.001	0.010
90	18	−0.536 (−2.262, 1.189)	0.542	97.78%	13.397	< 0.001	
NR	56	−2.391 (−2.905, −1.876)	< 0.001	91.10%	3.379	< 0.001	
Specimen type (AM GROUP)							
Specimen	115	−2.603 (−3.186, −2.020)	< 0.001	96.45%	9.534	< 0.001	NA
Denture resin specimen dimension							
10 × 10 × 3 mm	18	−7.573 (−8.313, −6.832)	< 0.001	35.45%	0.888	0.051	< 0.001
10 × 10 × 20 mm	3	−1.087 (−1.612, −0.563)	< 0.001	0.00%	0.000	0.621	
10 × 2 mm	14	−1.090 (−3.277, 1.097)	0.329	98.04%	16.776	< 0.001	
10 × 10 × 11 mm	2	−1.903 (−2.569, −1.236)	< 0.001	0.00%	0.000	0.591	
10 × 10 × 2.5 mm	4	−1.533 (−2.017, −1.050)	< 0.001	0.00%	0.000	0.819	
10 × 10 × 40 mm	12	−4.494 (−6.517, −2.472)	< 0.001	96.91%	11.938	< 0.001	
10 × 10 × 43 mm	9	0.164 (−1.437, 1.765)	0.841	94.31%	5.608	< 0.001	
20 × 10 × 3 mm	20	−2.496 (−3.158, −1.834)	< 0.001	78.92%	1.724	< 0.001	
25 × 25 × 3 mm	20	−1.262 (−1.717, −0.807)	< 0.001	76.01%	0.807	< 0.001	
36 × 7 mm	2	−1.323 (−3.158, 0.513)	0.158	85.68%	1.504	0.008	
7.5 × 2 mm	5	−1.387 (−1.894, −0.880)	< 0.001	28.86%	0.097	0.231	
8 × 8 × 8 mm	4	1.311 (0.689, 1.933)	< 0.001	41.79%	0.168	0.150	
80 × 7 × 2 mm	2	−2.823 (−5.563, −0.084)	0.043	88.71%	3.475	0.003	
Liner specimen dimension (AM GROUP)							
10 × 3 mm	20	−1.262 (−1.717, −0.807)	< 0.001	76.01%	0.807	< 0.001	< 0.001
10 × 10 × 3 mm	26	−2.240 (−3.541, −0.938)	0.001	97.18%	10.903	< 0.001	
3 × 5 mm	19	−1.198 (−2.725, 0.330)	0.124	97.34%	11.003	< 0.001	
3 × 7 × 2 mm	2	−2.823 (−5.563, −0.084)	0.043	88.71%	3.475	0.003	
3.6 × 3 mm	20	−2.496 (−3.158, −1.834)	< 0.001	78.92%	1.724	< 0.001	
4 × 3 mm	4	1.311 (0.689, 1.933)	< 0.001	41.79%	0.168	0.150	
4 × 6 mm	22	−6.553 (−7.854, −5.252)	< 0.001	90.69%	8.218	< 0.001	
7 × 3 mm	2	−1.323 (−3.158, 0.513)	0.158	85.68%	1.504	0.008	
Specimen shape (AM GROUP)							
Beam	28	−2.276 (−3.485, −1.067)	< 0.001	96.94%	10.109	< 0.001	0.037
Cylindrical	21	−1.217 (−2.575, 0.141)	0.079	97.04%	9.586	< 0.001	
Plate/Block + Cylindrical	66	−3.182 (−3.924, −2.441)	< 0.001	95.65%	8.707	< 0.001	
Milling machine brand							
CORiTEC 250i, imes‐icore	5	−1.387 (−1.894, −0.880)	< 0.001	28.86%	0.097	0.231	< 0.001
CORiTEC 550i, imes‐icore	14	−1.090 (−3.277, 1.097)	0.329	98.04%	16.776	< 0.001	
DWX‐52D, DGShape	4	1.311 (0.689, 1.933)	< 0.001	41.79%	0.168	0.150	
NR	59	−3.643 (−4.378, −2.908)	< 0.001	94.73%	7.498	< 0.001	
PrograMill PM7, Ivoclar	12	−4.494 (−6.517, −2.472)	< 0.001	96.91%	11.938	< 0.001	
Redon	2	−1.323 (−3.158, 0.513)	0.158	85.68%	1.504	0.008	
Roland DWX‐51D, Roland DG Corporation	2	−2.823 (−5.563, −0.084)	0.043	88.71%	3.475	0.003	
S2 Milling machine, VHF	8	−0.883 (−1.532, −0.233)	0.008	69.53%	0.606	0.003	
Zenotec Select, WielandDental	9	0.164 (−1.437, 1.765)	0.841	94.31%	5.608	< 0.001	
Number of milling axis							
5	54	−1.497 (−2.349, −0.645)	0.001	96.95%	9.697	< 0.001	< 0.001
NR	61	−3.559 (−4.275, −2.843)	< 0.001	94.77%	7.374	< 0.001	
Material (SM GROUP)							
PMMA‐based resin	115	−2.603 (−3.186, −2.020)	< 0.001	96.45%	9.534	< 0.001	NA
Material brand (SM GROUP)							
Anaxdent, Anaxdent	4	−1.308 (−2.237, −0.379)	0.006	73.39%	0.656	0.012	< 0.001
AvaDent, AvaDent Digital Dental Solutions	11	−6.377 (−8.146, −4.608)	< 0.001	89.43%	7.561	< 0.001	
IvoBase CAD, Ivoclar Vivadent	36	−3.135 (−4.312, −1.957)	< 0.001	97.00%	12.231	< 0.001	
Ivotion, Ivoclar Vivadent	1	−2.097 (−3.072, −1.123)	< 0.001	NA	0.000	NA	
L‐Temp, Degos Dental	10	−1.882 (−2.599, −1.165)	< 0.001	70.14%	0.919	< 0.001	
Luciton 199 Denture Base Disc, Dentsply Sirona	6	−4.726 (−8.477, −0.975)	0.014	98.26%	20.957	< 0.001	
Polident, Pearson Dental Supply	14	−1.090 (−3.277, 1.097)	0.329	98.04%	16.776	< 0.001	
Polident, Polident d.o.o.	4	−1.803 (−3.176, −0.429)	0.010	85.64%	1.663	< 0.001	
Smile Cam, Pressing Dental	12	−0.148 (−0.937, 0.640)	0.712	86.90%	1.679	< 0.001	
Temp Basic Tissue, Zirkonzahn	10	−3.206 (−4.308, −2.103)	< 0.001	80.93%	2.442	< 0.001	
Yamahachi, Yamahachi Dental MFG	7	−1.360 (−1.892, −0.827)	< 0.001	53.75%	0.276	0.044	
Specimen type (SM GROUP)							
Specimen	115	−2.603 (−3.186, −2.020)	< 0.001	96.45%	9.534	< 0.001	NA
Denture resin specimen dimension (SM GROUP)							
10 × 10 × 3 mm	18	−7.573 (−8.313, −6.832)	< 0.001	35.45%	0.888	0.051	< 0.001
10 ×10 × 20 mm	3	−1.087 (−1.612, −0.563)	< 0.001	0.00%	0.000	0.621	
10 × 2 mm	14	−1.090 (−3.277, 1.097)	0.329	98.04%	16.776	< 0.001	
10 × 10 × 11 mm	2	−1.903 (−2.569, −1.236)	< 0.001	0.00%	0.000	0.591	
10 × 10 × 2.5 mm	4	−1.533 (−2.017, −1.050)	< 0.001	0.00%	0.000	0.819	
10 × 10 × 40 mm	12	−4.494 (−6.517, −2.472)	< 0.001	96.91%	11.938	< 0.001	
10 × 10 × 43 mm	9	0.164 (−1.437, 1.765)	0.841	94.31%	5.608	< 0.001	
20 × 10 × 3 mm	20	−2.496 (−3.158, −1.834)	< 0.001	78.92%	1.724	< 0.001	
25 × 25 × 3 mm	20	−1.262 (−1.717, −0.807)	< 0.001	76.01%	0.807	< 0.001	
36 × 7 mm	2	−1.323 (−3.158, 0.513)	0.158	85.68%	1.504	0.008	
7.5 × 2 mm	5	−1.387 (−1.894, −0.880)	< 0.001	28.86%	0.097	0.231	
8 × 8 × 8 mm	4	1.311 (0.689, 1.933)	< 0.001	41.79%	0.168	0.150	
80 × 7 × 2 mm	2	−2.823 (−5.563, −0.084)	0.043	88.71%	3.475	0.003	
Liner specimen dimension (SM GROUP)							
10 × 10 × 3 mm	26	−2.240 (−3.541, −0.938)	0.001	97.18%	10.903	< 0.001	< 0.001
10 × 3 mm	20	−1.262 (−1.717, −0.807)	< 0.001	76.01%	0.807	< 0.001	
3 × 5 mm	19	−1.198 (−2.725, 0.330)	0.124	97.34%	11.003	< 0.001	
3 × 7 × 2 mm	2	−2.823 (−5.563, −0.084)	0.043	88.71%	3.475	0.003	
3.6 × 3 mm	20	−2.496 (−3.158, −1.834)	< 0.001	78.92%	1.724	< 0.001	
4 × 3 mm	4	1.311 (0.689, 1.933)	< 0.001	41.79%	0.168	0.150	
4 × 6 mm	22	−6.553 (−7.854, −5.252)	< 0.001	90.69%	8.218	< 0.001	
7 × 3 mm	2	−1.323 (−3.158, 0.513)	0.158	85.68%	1.504	0.008	
Specimen shape (SM GROUP)							
Beam	28	−2.276 (−3.485, −1.067)	< 0.001	96.94%	10.109	< 0.001	0.037
Cylindrical	21	−1.217 (−2.575, 0.141)	0.079	97.04%	9.586	< 0.001	
Plate/Block + Cylindrical	66	−3.182 (−3.924, −2.441)	< 0.001	95.65%	8.707	< 0.001	
Type of liner							
Hard	80	−2.813 (−3.575, −2.052)	< 0.001	96.59%	11.333	< 0.001	0.210
Soft	35	−2.106 (−2.907, −1.306)	< 0.001	95.17%	5.412	< 0.001	
Type of polymerization of liner							
Auto	112	−2.641 (−3.240, −2.041)	< 0.001	96.51%	9.819	< 0.001	0.040
Heat	3	−1.359 (−2.426, −0.292)	0.013	72.71%	0.645	0.025	
Liner material							
Acrylate‐based	100	−2.818 (−3.485, −2.151)	< 0.001	96.74%	10.876	< 0.001	0.002
Silicone‐based	15	−1.310 (−1.968, −0.653)	< 0.001	83.62%	1.401	< 0.001	
Liner brand							
COE Soft, GC America	6	−1.640 (−2.449, −0.831)	< 0.001	78.42%	0.793	0.001	< 0.001
GC RELINE, GC America	4	−1.533 (−2.017, −1.050)	< 0.001	0.00%	0.000	0.819	
GC Soft Liner, GC Corp.	8	−0.563 (−0.873, −0.253)	< 0.001	0.00%	0.000	1.000	
Kooliner, GC America	1	−0.845 (−1.725, 0.034)	0.060	NA	0.000	NA	
Lucitone 199 denture base material, Dentsply Sirona	1	−1.496 (−2.455, −0.537)	0.002	NA	0.000	NA	
Lynal, Dentsply Sirona	6	−7.436 (−9.610, −5.263)	< 0.001	83.45%	5.927	< 0.001	
Major repair, Major Prodotti Dentari SPA	18	−7.573 (−8.313, −6.832)	< 0.001	35.45%	0.888	0.051	
Meliodent, Kulzer	5	−1.387 (−1.894, −0.880)	< 0.001	28.86%	0.097	0.231	
Molloplast‐B, Detax	2	−1.323 (−3.158, 0.513)	0.158	85.68%	1.504	0.008	
PalaXpress, Kulzer	3	−2.348 (−3.666, −1.030)	< 0.001	69.24%	0.932	0.042	
Paladur, Kulzer	4	−2.031 (−7.411, 3.350)	0.459	98.37%	29.278	< 0.001	
Palapress, Kulzer	20	−2.496 (−3.158, −1.834)	< 0.001	78.92%	1.724	< 0.001	
ProBase Cold, Ivoclar Vivadent	4	−2.164 (−3.418, −0.910)	0.001	82.66%	1.325	0.005	
Reline II soft, GC Europe	6	−2.551 (−3.024, −2.078)	< 0.001	0.00%	0.000	0.803	
Sofreliner Tough M, Tokuyama Dental Corp.	2	−0.200 (−0.857, 0.457)	0.551	0.00%	0.000	0.953	
Tokuyama Rebase II, Tokuyama Dental Corp.	3	−1.856 (−2.489, −1.223)	< 0.001	0.00%	0.000	0.477	
Ufi Gel P, VOCO	2	−0.869 (−2.861, 1.124)	0.393	86.42%	1.788	0.007	
Ufi Gel SC, VOCO	3	0.044 (−0.707, 0.795)	0.908	47.13%	0.208	0.151	
Ufi Gel hard C, VOCO	13	0.083 (−1.959, 2.126)	0.936	97.57%	13.558	< 0.001	
Unifast Trad, GC America	4	1.311 (0.689, 1.933)	< 0.001	41.79%	0.168	0.150	
Surface preparation of denture resin							
Chemical treatment	19	−3.719 (−5.916, −1.522)	0.001	97.12%	22.645	< 0.001	0.095
Mechanical and chemical treatment	6	−1.835 (−3.015, −0.656)	0.002	83.87%	1.787	< 0.001	
Mechanical treatment	22	−3.340 (−5.019, −1.660)	< 0.001	97.59%	15.273	< 0.001	
No treatment	66	−2.173 (−2.772, −1.575)	< 0.001	94.80%	5.682	< 0.001	
Physicochemical treatment	2	−1.155 (−2.143, −0.166)	0.022	55.92%	0.285	0.132	
Bonding agent (AM GROUP)							
Bonding adhesive	27	−0.517 (−1.459, 0.425)	0.282	95.51%	5.858	< 0.001	< 0.001
Methacrylate monomer	20	−4.094 (−5.343, −2.846)	< 0.001	93.18%	7.227	< 0.001	
NR	3	−1.087 (−1.612, −0.563)	< 0.001	0.00%	0.000	0.621	
No bonding agent	65	−3.120 (−3.901, −2.338)	< 0.001	96.31%	9.618	< 0.001	
Bonding agent (SM GROUP)							
Bonding adhesive	25	−0.333 (−1.305, 0.638)	0.501	95.52%	5.760	< 0.001	< 0.001
Methacrylate monomer	22	−3.957 (−5.099, −2.816)	< 0.001	92.91%	6.620	< 0.001	
NR	3	−1.087 (−1.612, −0.563)	< 0.001	0.00%	0.000	0.621	
No bonding agent	65	−3.120 (−3.901, −2.338)	< 0.001	96.31%	9.618	< 0.001	
Evaluation test							
Shear bond strength	67	−3.211 (−4.100, −2.321)	< 0.001	96.68%	12.962	< 0.001	0.018
Tensile bond strength	48	−1.810 (−2.562, −1.058)	< 0.001	95.85%	6.642	< 0.001	

Abbreviations: AM GROUP, additive manufacturing group; CI, confidence interval; DLP, digital Light processing; DLS, digital light synthesis; *I* ^2^, percentage of total variation across studies due to heterogeneity; LCD, liquid crystal display; *N*, number of included comparisons; NA, not applicable; NR, not reported; *p* value, probability value for statistical significance of SMD; *p* diff, *p* value for difference between subgroups; *p* (heterogeneity), PMMA, polymethyl methacrylate; *p* value assessing heterogeneity; SLA, stereolithography; SMD, standardized mean difference; SM GROUP, subtractive manufacturing group; τ², Between‐study variance in random‐effects meta‐analysis.

### 3.4 Publication Bias

Publication‐bias diagnostics indicated small‐study effects, as reflected by significant Egger's and Begg's test results and the asymmetry observed in the funnel plots. These findings suggest that the available evidence may be influenced by selective reporting or underrepresentation of null results, which in practice could lead to an overestimation of bonding performance in smaller studies. However, the trim‐and‐fill analyses did not impute any missing studies for either tooth–base or reline–base outcomes; therefore, the adjusted funnel plots would be identical to the originals and were not reported separately. Despite this, given the high heterogeneity among the included studies, the possibility of publication bias cannot be definitively excluded.

### 3.5 Risk of Bias

All 20 in‐vitro studies met 55%–70% of the QUIN criteria, so every study was classified as medium risk of bias (Table 4). The average score was 62.8%. Nine studies scored 60%, six reached 70%, two scored 65%, and three fell to 55%. The domains most frequently under‐reported were Operator details (C6)—scored “0” in every study—and Blinding of outcome assessment (C10), which was absent in all reports. Sample‐size calculations (C2) were omitted in over half of the papers, and no study provided randomization details (C7, not applicable in this experimental context).

**Table 4:** Evaluation of the quality of the included studies using the QUIN Tool's grading system.

References	C1	C2	C3	C4	C5	C6	C7	C8	C9	C10	C11	C12	Total score	Risk of bias calculation (%)	Risk of bias interpretation
Kane and Shah (2023)	2	2	NA	1	2	0	NA	2	0	0	1	2	12	60	Medium risk
Choi et al. (2020)	2	0	NA	2	2	0	NA	2	0	0	2	2	12	60	Medium risk
A. Mohamed et al. (2023a)	2	2	NA	2	2	0	NA	2	0	0	2	2	14	70	Medium risk
Alanazi et al. (2024)	2	1	NA	2	1	0	NA	2	0	0	2	2	12	60	Medium risk
Löscher et al. (2024)	1	0	NA	2	2	0	NA	2	0	0	2	2	11	55	Medium risk
Alfaraj et al. (2023)	2	0	NA	2	2	0	NA	2	0	0	2	2	12	60	Medium risk
Vuksic et al. (2023)	2	0	NA	2	2	0	NA	2	0	0	2	2	12	60	Medium risk
Wemken et al. (2021)	2	0	NA	2	2	0	NA	2	0	0	2	2	12	60	Medium risk
Gad et al. (2022)	2	2	NA	2	2	0	NA	2	0	0	2	2	14	70	Medium risk
Awad et al. (2023)	2	0	NA	1	2	0	NA	2	0	0	2	2	11	55	Medium risk
Gibreel et al. (2024)	2	0	NA	2	2	0	NA	2	0	0	2	2	12	60	Medium risk
Sahin et al. (2024b)	2	2	NA	2	2	0	NA	2	0	0	2	2	14	70	Medium risk
Karaokutan and Ayvaz (2025)	2	2	NA	2	2	0	NA	2	0	0	2	2	14	70	Medium risk
Mert et al. (2023)	2	2	NA	1	2	0	NA	2	0	0	2	2	13	65	Medium risk
Albazroun et al. (2024)	2	2	NA	2	2	0	NA	2	0	0	2	2	14	70	Medium risk
Karaokutan et al. (2024)	2	0	NA	2	2	0	NA	2	0	0	2	2	12	60	Medium risk
Htat et al. (2024)	1	2	NA	2	2	0	NA	2	0	0	2	2	13	65	Medium risk
Janyaprasert et al. (2024)	2	0	NA	2	2	0	NA	2	0	0	2	2	12	60	Medium risk
Tugut et al. (2024)	2	0	NA	2	1	0	NA	2	0	0	2	2	11	55	Medium risk
Li et al. (2025)	2	2	NA	2	2	0	NA	2	0	0	2	2	14	70	Medium risk

*Note:* Assessment criteria; C1, clearly stated aims/objectives; C2, detailed explanation of sample size calculation; C3, detailed explanation of sampling technique; C4, details of comparison group; C5, detailed explanation of methodology; C6, operator details; C7, randomization; C8, method of measurement of outcome; C9, Outcome assessor details; C10, blinding; C11, Statistical analysis; C12, Presentation of results. Scoring; 2, adequately specified; 1, inadequately specified; 0, not specified; NA, not applicable.

### 3.6 Certainty of Evidence

The GRADE assessment rated the certainty of evidence as low for the tooth–base adhesion outcome, primarily due to risk of bias and inconsistency across studies, and very low for the reline–base adhesion outcome, with additional downgrades for imprecision and suspected publication bias (Table 5).

**Table 5:** GRADE certainty‐of‐evidence assessment for included outcomes.

Outcome	No. of studies (comparisons)	Risk of bias	Inconsistency	Indirectness	Imprecision	Publication bias	Overall certainty of evidence	Comments
Tooth–base bond strength (AM vs. SM)	5 (39)	Serious (limitations in randomization, blinding, and reporting in several in vitro studies)	Serious (heterogeneity in testing methods and materials)	Not serious (direct relevance to the review question)	Not serious (number of comparisons adequate for in vitro evidence)	Possible (funnel plot asymmetry observed, no studies imputed by trim‐and‐fill)	Low	Downgraded for risk of bias and inconsistency
Reline–base bond strength (AM vs. SM)	15 (115)	Serious (lack of blinding, methodological variability)	Serious (differences in relining materials and protocols)	Not serious	Serious (wide confidence intervals in several studies)	Possible (funnel plot asymmetry observed, no studies imputed by trim‐and‐fill)	Very low	Downgraded for risk of bias, inconsistency, imprecision, and suspected publication bias

## 4 Discussion

The pooled evidence showed that denture teeth and reline materials bond much more effectively to SM bases than to AM ones. After sensitivity checks, tooth‐to‐base strength was clearly higher for milled PMMA, and reline‐to‐base strength was markedly greater as well, with both effects remaining stable when any individual comparison was removed and still evident after accounting for possible publication bias. These outcomes demonstrate that the two fabrication routes are not equivalent, so the study's null hypothesis of “no difference between AM and SM bonding performance” is rejected for both interfaces.

The superior bonding of milled PMMA originates from its industrial polymerization protocol. Prolonged heat‐and‐pressure curing yields a densely cross‐linked, homogeneous matrix with a high degree of conversion, low residual monomer, and an elevated glass transition temperature (Zafar 2020). The surface retains pendant methacrylate groups, which, when activated by MMA primers, allow deep monomer penetration and covalent copolymerization, forming a semi‐interpenetrating polymer network resistant to hydrolytic degradation (Erbulak and Ergun 2023; Neto et al. 2024). The relatively polar surface of milled PMMA also promotes superior wettability, enhancing primer spreading and diffusion. In contrast, AM photopolymers are produced by the layer‐wise curing of dimethacrylate oligomers at ambient conditions using DLP, SLA, LCD, or related technologies. These materials exhibit lower conversion, reduced cross‐link density, oxygen‐inhibited surface layers, and microscopically stepped “stair‐lines” that concentrate stress (Chekkaramkodi et al. 2024; Rooney et al. 2024). The resulting substrate is chemically less polar, mechanically anisotropic, and dotted with voids: factors that hinder primer wetting and restrict radical grafting across the interface (Arias‐Ferreiro 2022). Such interfacial deficiencies explain why printed bases, especially those fabricated with coarse layer thicknesses and horizontal (0°) build orientations, consistently exhibited the greatest loss of bond strength (Dimitrova et al. 2023; Liang et al. 2025). Specimen geometry further influenced adhesion. Beam‐, cylindrical‐, and denture‐shaped specimens expose larger peripheral stair‐step defects, generating stress concentrations that accelerate crack initiation and adhesive failure. By contrast, flat block specimens, with fewer geometric irregularities, demonstrated higher bonding reliability (Farkas et al. 2023). On the tooth side, adhesion was further compromised when high‐cross‐linked or filler‐enriched PMMA teeth were bonded to AM bases, as the rigid polymeric networks restricted interfacial swelling and monomer diffusion, leading to brittle interfacial zones prone to early debonding. An instructive exception was fused deposition modeling (FDM) of PMMA. The extrusion of pre‐polymerized PMMA filaments at elevated temperature produced a surface comparable to conventionally processed pucks, with reduced voids and enhanced chain fusion. This processing route resulted in bonding strength equal to, or surpassing, that of milled PMMA (Yousfi et al. 2021). The finding underscores that adhesive performance is governed more by polymer chemistry and processing conditions than by whether a workflow is designated as additive or subtractive.

Successful relining depends on the ability of reline monomers to diffuse into the denture base, swell the polymer matrix, and copolymerize to form a semi‐interpenetrating network (Koseoglu et al. 2023). The industrially processed surface of milled PMMA, characterized by high conversion, abundant pendant methacrylate groups, and moderate solvent uptake capacity, provides an ideal substrate for this process. Both acrylic and silicone liners—regardless of hardness, polymerization mode, or application after minor surface roughening—can establish stable covalent linkages and mechanical interlocking with milled PMMA, thereby producing reliable adhesion (Zafar 2020). The polar nature of the surface further promotes wettability and homogeneous primer spreading, facilitating deep penetration of the reline monomer. Printed resins, in contrast, encounter several fundamental barriers to effective relining (Sturzenegger et al. 2025). The layer‐wise photopolymerization of dimethacrylate oligomers yields matrices that are more rigid, less polar, and deficient in pendant methacrylate sites, while also absorbing significantly less solvent (Abu‐elenain et al. 2013). These limitations restrict swelling and hinder monomer infiltration, leading to a shallow interdiffusion zone and fragile bonding. Moreover, residual photoinitiators and unreacted oligomers can quench radical activity during liner polymerization, reducing copolymerization efficiency and leaving weak, chemically unstable interfaces (Bayraktar et al. 2022). Interlayer valleys intrinsic to additive manufacturing further concentrate stresses, particularly peel forces generated during flexure of soft liners, accelerating interfacial crack initiation and propagation. The adhesive performance of AM bases was consistently weaker across liner types, hardness categories, and curing methods, confirming that the deficit originates from substrate chemistry rather than liner composition. While the use of methacrylate primers modestly improved adhesion, they failed to eliminate the performance gap, indicating that limited diffusion depth and poor chemical affinity remain the principal constraints (Bourgi et al. 2024). Printing parameters exacerbated these limitations. Light‐based AM technologies (SLA, DLP, LCD), especially at fine layer thicknesses with horizontal build orientations, generated surfaces with low polarity and internal porosity, both of which restrict primer infiltration and monomer diffusion (Chen and Wei 2025). The anisotropic microstructure of such builds creates preferential fracture planes at layer boundaries, further compromising bond stability under repetitive functional loading. This chemical explanation aligns with the mechanical one: printed interfaces are not only less receptive to monomer penetration but also more prone to stress concentration and crack propagation during mastication or liner flexure (Azab et al. 2025; Bourgi et al. 2024). Stair‐step defects at layer boundaries act as initiation sites for adhesive failure, while voids within incompletely cured regions reduce fracture toughness. Peel stresses, which are particularly relevant in soft silicone liners subjected to repeated flexure, are poorly resisted by AM bases due to their anisotropic structure. In contrast, the homogeneous microstructure of milled PMMA distributes stresses more evenly, limiting crack initiation and propagation under occlusal or relining loads. Taken together, these mechanistic factors explain the consistently inferior reline adhesion observed for AM denture bases. Clinically, such deficiencies suggest a higher likelihood of liner debonding, recurrent repairs, and reduced prosthesis longevity compared with milled counterparts, which may offset the initial advantages of rapid fabrication and digital customization.

Subgroup analyses indicated that bond strengths for AM denture bases were consistently reduced when printed with DLP or SLA technologies, when 50–100 µm layers were deposited, and when the build orientation was 0°. The use of methacrylate‐monomer primers or the absence of bonding agents further weakened adhesion compared with SM. In contrast, SM bases demonstrated stable adhesion across all conditions, highlighting the robustness of PMMA discs against variations in bonding protocols. These findings emphasize the critical role of polymerization kinetics, build orientation, and resin chemistry in determining adhesive reliability. A notable exception was fused deposition modeling (FDM) of PMMA filaments, which outperformed milled PMMA. The extrusion process at elevated temperatures appears to generate a surface morphology comparable to conventionally processed PMMA, with fewer interfacial defects and a higher degree of polymer fusion. This observation highlights that additive versus subtractive classification alone is insufficient to predict bonding potential; material chemistry and processing route are more decisive factors.

From a clinical perspective, these findings indicate that milled dentures remain the safest option when long‐term tooth retention and predictable chair‐side relining are paramount. Practitioners who nevertheless choose 3D printing for its speed or design freedom should be prepared to offset the material's intrinsic limitations by selecting resins with documented high conversion, orienting builds away from the horizontal plane to minimize staircase defects, combining vigorous mechanical roughening with a chemistry‐matched primer, and planning closer follow‐up for early rebonding or relining, especially when using soft silicone materials whose low modulus exposes the adhesive interface to repetitive peel stresses. The superior bonding of SM bases reduces the likelihood of debonding, rebonding procedures, or frequent relines, thereby decreasing maintenance costs and chairside time. In contrast, AM dentures may require earlier intervention, potentially offsetting the initial advantages of faster fabrication and lower material waste. Furthermore, repeated relining procedures can contribute to increased stresses on the residual ridge and may accelerate bone resorption, compromising long‐term oral health outcomes.

It should be noted that the heterogeneity of the included studies was very high in several analyses (*I* ^2^ > 90%). Such heterogeneity is not unexpected in systematic reviews of in vitro data, given the wide variability in experimental designs, denture base and liner/tooth materials, surface treatments, specimen geometries, and testing protocols. Nonetheless, this high level of inconsistency reduces the precision of pooled effect estimates and indicates that the quantitative values should be interpreted with caution. Rather than providing exact predictive figures, the meta‐analytic outcomes should be considered as reflecting overall performance trends, with subgroup analyses offering further insight into the factors contributing to variability. The criteria for outlier exclusion were based on influence diagnostics from leave‐one‐out sensitivity analyses (Viechtbauer and Cheung 2010), excluding the comparisons that were identified as disproportionately inflating heterogeneity. Their exclusion markedly reduced τ^2^ values and shifted the pooled tooth–base estimate from nonsignificant to significantly favoring milled bases. Emphasizing this contrast demonstrates both the robustness of the findings and the critical influence of individual datasets on pooled outcomes.

Despite consistent trends, several limitations must be considered. All included studies were laboratory‐based and relied on static shear or tensile protocols that cannot replicate thermal cycling, enzymatic degradation, or multidirectional occlusal loading in vivo; methodological heterogeneity remained high despite subgroup modeling; and a modest publication bias was detected, suggesting that some null or negative data may be missing from the record. The certainty‐of‐evidence assessment further indicated that the strength of the current evidence is limited, with low certainty for tooth–base adhesion and very low certainty for reline–base adhesion. These findings highlight the need for well‐designed, standardized studies with larger sample sizes to increase the reliability of future systematic reviews in this field. Moreover, denture resins evolve rapidly, so conclusions drawn from today's photopolymers may need revision as higher conversion, more polar printable materials—and extrusion‐based PMMA systems—reach the market.

Future research should focus on standardizing laboratory protocols, incorporating fatigue‐based testing, and conducting well‐controlled clinical trials to validate these findings under real‐world conditions. Rapid advances in printable resin formulations, including high‐conversion, more polar photopolymers and extrusion‐based PMMA systems, may alter the current evidence landscape. Continuous reassessment will therefore be essential to determine whether AM can achieve bonding reliability comparable to SM in complete denture fabrication.

## 5 Conclusions

Within the limitations of this systematic review, the following conclusions can be drawn from the available in vitro evidence:

1. Denture teeth generally exhibited stronger and more consistent bonding to milled PMMA bases than to additively manufactured bases.
2. Relining materials, irrespective of their composition or polymerization mode, also tended to adhere more effectively to milled denture bases compared with printed ones.
3. These findings suggest that subtractive manufacturing currently provides more reliable adhesion under laboratory conditions, while the clinical significance of these differences remains to be established.

## Author Contributions

**Safoura Ghodsi:** conceptualization, validation, resources, writing – review and editing, supervision, project administration. **Seyed Ali Mosaddad:** conceptualization, methodology, validation, software, resources, writing – review and editing, supervision, project administration. **Sarah Arzani:** methodology, software, investigation, data curation, writing – original draft. **Erfan Khorasani:** formal analysis, investigation, data curation, writing – original draft. Aida Mokhlesi: formal analysis, investigation, data curation, writing – original draft. **Shima Azadian:** software, visualization, data curation. All authors have read and approved the published version of the manuscript.

## Ethics Statement

The authors have nothing to report.

## Consent

The authors have nothing to report.

## Conflicts of Interest

The authors declare no conflicts of interest.

## Supporting information


**Supplementary Figure 1:** Leave‐one‐out sensitivity analysis for tooth–base bonding comparisons before removing the outliers. **Supplementary Figure 2:** Leave‐one‐out sensitivity analysis for tooth–base bonding comparisons after removing the outliers. **Supplementary Table 1:** Detailed search queries and methodologies used across databases to retrieve relevant records. **Supplementary Table 2:** Subgroup meta‐analysis results for tooth–base bond strength comparisons between additive and subtractive denture bases. **Supplementary Table 3:** Subgroup meta‐analysis results for reline–base bond strength comparisons between additive and subtractive denture bases.

## Data Availability

The data presented in this study are available on request from the corresponding author.
