# Supplementary material for: How Does the Bonding Strength of Reline Materials and Denture Teeth Vary Between 3D‐Printed and Milled Complete Denture Bases? A Systematic Review and Meta‐Analysis
Source: Clin Exp Dent Res. 2025 Oct 7;11(5):e70234. doi: 10.1002/cre2.70234 (PMC12502631; doi:10.1002/cre2.70234)
Supplement: Supplementary file 1 — Supplementary Figure 1: Leave‐one‐out sensitivity analysis for tooth–base bonding comparisons before removing the outliers. Supplementary Figure 2: Leave‐one‐out sensitivity analysis for tooth–base bonding comparisons after removing the outliers. Supplementary Table 1: Detailed search queries and methodologies used across databases to retrieve relevant records. Supplementary Table 2: Subgroup meta‐analysis results for tooth–base bond strength comparisons between additive and subtractive denture bases. Supplementary Table 3: Subgroup meta‐analysis results for reline–base bond strength comparisons between additive and subtractive denture bases. [file CRE2-11-e70234-s001.docx]

**Supplementary Table 1.** Detailed search queries and methodologies used across databases to retrieve relevant records

| **Database** | **Database-specified search query** |
| --- | --- |
| **PubMed** | (Dentures[Mesh Terms] OR Dentures[Title/Abstract] OR Denture[Title/Abstract] OR "Denture, Complete"[Mesh Terms] OR "Complete Denture"[Title/Abstract] OR "Complete Dentures"[Title/Abstract] OR "Denture, Complete, Lower"[Mesh Terms] OR "Denture, Complete, Upper"[Mesh Terms] OR "complete removable denture"[Title/Abstract] OR "complete removable dentures"[Title/Abstract] OR "complete removable dental prostheses"[Title/Abstract] OR "complete dental prostheses"[Title/Abstract] OR "full denture"[Title/Abstract] OR "removable dental prostheses"[Title/Abstract] OR "removable denture"[Title/Abstract] OR "removable dentures"[Title/Abstract] OR "total denture"[Title/Abstract] OR "total dentures"[Title/Abstract] OR "total dental prostheses"[Title/Abstract] OR "total removable denture"[Title/Abstract] OR "total removable dental prostheses"[Title/Abstract]) AND ("Printing, Three-Dimensional"[Mesh Terms] OR "Three-Dimensional Printings"[Title/Abstract] OR "3-Dimensional Printing"[Title/Abstract] OR "3 Dimensional Printing"[Title/Abstract] OR "3-Dimensional Printings"[Title/Abstract] OR "3-D Printing"[Title/Abstract] OR "3 D Printing"[Title/Abstract] OR "3-D Printings"[Title/Abstract] OR "Three-Dimensional Printing"[Title/Abstract] OR "Three Dimensional Printing"[Title/Abstract] OR "3D Printing"[Title/Abstract] OR "3D Printings"[Title/Abstract] OR Stereolithography[Mesh Terms] OR Stereolithography[Title/Abstract] OR Stereolithographie[Title/Abstract] OR "additive manufacturing"[Title/Abstract] OR "additive fabrication"[Title/Abstract] OR "rapid prototyping"[Title/Abstract] OR "3D printing method"[Title/Abstract] OR "3d printed denture"[Title/Abstract] OR "layered manufacturing"[Title/Abstract] OR "solid freeform fabrication"[Title/Abstract] OR "direct digital manufacturing"[Title/Abstract] OR "selective laser sintering"[Title/Abstract] OR "electron beam melting"[Title/Abstract] OR "Electron-beam additive manufacturing"[Title/Abstract] OR "Additive technology"[Title/Abstract] OR "3d printed"[Title/Abstract] OR "3d-printed"[Title/Abstract] OR "3-D Printed"[Title/Abstract] OR "Three-Dimensional Printed"[Title/Abstract] OR "3-Dimensional Printed"[Title/Abstract] OR "3 Dimensional Printed"[Title/Abstract]) AND (Mill*[Title/Abstract] OR "Subtractive Manufacturing"[Title/Abstract] OR "computer-aided milling"[Title/Abstract] OR "CAD/CAM milling"[Title/Abstract] OR CAD/CAM[Title/Abstract] OR "digital milling"[Title/Abstract] OR "precision milling"[Title/Abstract] OR "subtractive fabrication"[Title/Abstract] OR "CNC machining"[Title/Abstract]) |
| **Scopus** | TITLE-ABS-KEY(dentures OR denture OR "Complete Denture" OR "Complete Dentures" OR "complete removable denture" OR "complete removable dentures" OR "complete removable dental prostheses" OR "complete dental prostheses" OR "full denture" OR "removable dental prostheses" OR "removable denture" OR "removable dentures" OR "total denture" OR "total dentures" OR "total dental prostheses" OR "total removable denture" OR "total removable dental prostheses") AND TITLE-ABS-KEY("Three-Dimensional Printings" OR "3-Dimensional Printing" OR "3 Dimensional Printing" OR "3-Dimensional Printings" OR "3-D Printing" OR "3 D Printing" OR "3-D Printings" OR "Three-Dimensional Printing" OR "Three Dimensional Printing" OR "3D Printing" OR "3D Printings" OR stereolithography OR stereolithographie OR "additive manufacturing" OR "additive fabrication" OR "rapid prototyping" OR "3D printing method" OR "3d printed denture" OR "layered manufacturing" OR "solid freeform fabrication" OR "direct digital manufacturing" OR "selective laser sintering" OR "electron beam melting" OR "Electron-beam additive manufacturing" OR "Additive technology" OR "3d printed" OR "3d-printed" OR "3-D Printed" OR "Three-Dimensional Printed" OR "3-Dimensional Printed" OR "3 Dimensional Printed") AND TITLE-ABS-KEY(mill* OR "Subtractive Manufacturing" OR "computer-aided milling" OR "CAD/CAM milling" OR cad/cam OR "digital milling" OR "precision milling" OR "subtractive fabrication" OR "CNC machining") |
| **Embase** | (dentures:ti,ab,kw OR denture:ti,ab,kw OR 'complete denture':ti,ab,kw OR 'complete dentures':ti,ab,kw OR 'complete removable denture':ti,ab,kw OR 'complete removable dentures':ti,ab,kw OR 'complete removable dental prostheses':ti,ab,kw OR 'complete dental prostheses':ti,ab,kw OR 'full denture':ti,ab,kw OR 'removable dental prostheses':ti,ab,kw OR 'removable denture':ti,ab,kw OR 'removable dentures':ti,ab,kw OR 'total denture':ti,ab,kw OR 'total dentures':ti,ab,kw OR 'total dental prostheses':ti,ab,kw OR 'total removable denture':ti,ab,kw OR 'total removable dental prostheses':ti,ab,kw) AND ('three-dimensional printings':ti,ab,kw OR '3-dimensional printing':ti,ab,kw OR '3 dimensional printing':ti,ab,kw OR '3-dimensional printings':ti,ab,kw OR '3-d printing':ti,ab,kw OR '3 d printing':ti,ab,kw OR '3-d printings':ti,ab,kw OR 'three-dimensional printing':ti,ab,kw OR 'three dimensional printing':ti,ab,kw OR '3d printing':ti,ab,kw OR '3d printings':ti,ab,kw OR stereolithography:ti,ab,kw OR stereolithographie:ti,ab,kw OR 'additive manufacturing':ti,ab,kw OR 'additive fabrication':ti,ab,kw OR 'rapid prototyping':ti,ab,kw OR '3d printing method':ti,ab,kw OR '3d printed denture':ti,ab,kw OR 'layered manufacturing':ti,ab,kw OR 'solid freeform fabrication':ti,ab,kw OR 'direct digital manufacturing':ti,ab,kw OR 'selective laser sintering':ti,ab,kw OR 'electron beam melting':ti,ab,kw OR 'electron-beam additive manufacturing':ti,ab,kw OR 'additive technology':ti,ab,kw OR '3d printed':ti,ab,kw OR '3d-printed':ti,ab,kw OR '3-d printed':ti,ab,kw OR 'three-dimensional printed':ti,ab,kw OR '3-dimensional printed':ti,ab,kw OR '3 dimensional printed':ti,ab,kw) AND (mill*:ti,ab,kw OR 'subtractive manufacturing':ti,ab,kw OR 'computer-aided milling':ti,ab,kw OR 'cad cam milling':ti,ab,kw OR 'cad cam':ti,ab,kw OR 'digital milling':ti,ab,kw OR 'precision milling':ti,ab,kw OR 'subtractive fabrication':ti,ab,kw OR 'cnc machining':ti,ab,kw) |
| **Web of Science** | TS=(Dentures OR Denture OR "Complete Denture" OR "Complete Dentures" OR "complete removable denture" OR "complete removable dentures" OR "complete removable dental prostheses" OR "complete dental prostheses" OR "full denture" OR "removable dental prostheses" OR "removable denture" OR "removable dentures" OR "total denture" OR "total dentures" OR "total dental prostheses" OR "total removable denture" OR "total removable dental prostheses") AND TS=("Three-Dimensional Printings" OR "3-Dimensional Printing" OR "3 Dimensional Printing" OR "3-Dimensional Printings" OR "3-D Printing" OR "3 D Printing" OR "3-D Printings" OR "Three-Dimensional Printing" OR "Three Dimensional Printing" OR "3D Printing" OR "3D Printings" OR Stereolithography OR Stereolithographie OR "additive manufacturing" OR "additive fabrication" OR "rapid prototyping" OR "3D printing method" OR "3d printed denture" OR "layered manufacturing" OR "solid freeform fabrication" OR "direct digital manufacturing" OR "selective laser sintering" OR "electron beam melting" OR "Electron-beam additive manufacturing" OR "Additive technology" OR "3d printed" OR "3d-printed" OR "3-D Printed" OR "Three-Dimensional Printed" OR "3-Dimensional Printed" OR "3 Dimensional Printed") AND TS=(Mill* OR "Subtractive Manufacturing" OR "computer-aided milling" OR "CAD/CAM milling" OR 'CAD CAM' OR "digital milling" OR "precision milling" OR "subtractive fabrication" OR "CNC machining") |
| **The Cochrane Library** | \| #1 \| MeSH descriptor: [Dentures] in all MeSH products \| 1235 \| \| --- \| --- \| --- \| \| #2 \| MeSH descriptor: [Denture, Complete] explode all trees \| 505 \| \| #3 \| MeSH descriptor: [Denture, Complete, Lower] explode all trees \| 203 \| \| #4 \| MeSH descriptor: [Denture, Complete, Upper] explode all trees \| 97 \| \| #5 \| (Dentures OR Denture OR "Complete Denture" OR "Complete Dentures" OR "complete removable denture" OR "complete removable dentures" OR "complete removable dental prostheses" OR "complete dental prostheses" OR "full denture" OR "removable dental prostheses" OR "removable denture" OR "removable dentures" OR "total denture" OR "total dentures" OR "total dental prostheses" OR "total removable denture" OR "total removable dental prostheses"):ti,ab,kw \| 2896 \| \| #6 \| MeSH descriptor: [Printing, Three-Dimensional] explode all trees \| 201 \| \| #7 \| MeSH descriptor: [Stereolithography] explode all trees \| 5 \| \| #8 \| ("Three-Dimensional Printings" OR "3-Dimensional Printing" OR "3 Dimensional Printing" OR "3-Dimensional Printings" OR "3-D Printing" OR "3 D Printing" OR "3-D Printings" OR "Three-Dimensional Printing" OR "Three Dimensional Printing" OR "3D Printing" OR "3D Printings" OR Stereolithography OR Stereolithographie OR "additive manufacturing" OR "additive fabrication" OR "rapid prototyping" OR "3D printing method" OR "3d printed denture" OR "layered manufacturing" OR "solid freeform fabrication" OR "direct digital manufacturing" OR "selective laser sintering" OR "electron beam melting" OR "Electron-beam additive manufacturing" OR "Additive technology" OR "3d printed" OR "3d-printed" OR "3-D Printed" OR "Three-Dimensional Printed" OR "3-Dimensional Printed" OR "3 Dimensional Printed"):ti,ab,kw \| 1050 \| \| #9 \| (Mill* OR "Subtractive Manufacturing" OR "computer-aided milling" OR "CAD CAM milling" OR "CAD CAM" OR "digital milling" OR "precision milling" OR "subtractive fabrication" OR "CNC machining"):ti,ab,kw \| 44800 \| \| #10 \| (#1 OR #2 OR #3 OR #4 OR #5) AND (#6 OR #7 OR #8) AND #9 \| 18 \| |
| **Google Scholar** | (Dentures OR Denture OR "Complete Denture" OR "Complete Dentures") AND ("Three-Dimensional Printings" OR "3-D Printing" OR "3 D Printing" OR "3-D Printings" OR "3D Printing" OR "3D Printings") AND (Mill* OR "Subtractive Manufacturing") |

**Supplementary Table 2.** Subgroup meta-analysis results for tooth–base bond strength comparisons between additive and subtractive denture bases.

| **Author/ Year/Country/ Reference** | **Study model/ digitalization method/ digitalization model and brand/ design software** | **3D-printing technology/ brand/ material/ material brand/ layer thickness/ build orientation/ specimen type/ specimen dimension/ specimen type/ specimen dimension/ specimen shape/ post-processing approach/ bonded teeth material/ teeth chemical composition/ teeth dimension/ teeth shape/ bonding agent/ teeth pretreatment** | **Milling machine setting/ brand/ material/ material brand/ specimen type/ specimen dimension/ specimen shape/ post-processing approach/ / bonded teeth material/ teeth chemical composition/ teeth dimension/ teeth shape/ bonding agent/ teeth pretreatment** | **Total sample size (AM/ SM)** | **Aging Process/ aging duration (month)/ thermocycling cycles/ evaluation test/ test machine/ test machine brand/ Crosshead speed (mm/min)** | **Reported values for the studied measurement outcomes (mean ± SD) for AM denture teeth bond strength (MPa)** | **Reported values for studied measurement outcomes (mean ± SD) for SM denture teeth bond strength (MPa)** | **Conclusion(s)** |
| --- | --- | --- | --- | --- | --- | --- | --- | --- |
| Kane et al./ 2023/ USA/ (Kane and Shah, 2023) | Intaglio surface of denture tooth/ Extraoral scanner/ D750, 3Shape/ Meshmixer, Autodesk | T1: SLA, T2: DLS/ B1: Form 2, Formlabs, B2: M2, Carbon/ non-PMMA resin/ MB1: Denture Base Resin, Formlabs, MB2: Lucitone Digital Print, Dentsply Sirona/ L1: 50, L2: 100/ NR/ Specimen/ NR/ Beam/ NR/ PMMA based resin/ Microfiller reinforced polymer matrix composite/ VITA Vitapan XL T44, #8, VITA Zahnfabrik/ NR/ Tooth shape/ Ivobase CAD bonding system, Ivoclar/ Mechanical treatment | 5-axis/ VersaMil Inc./ PMMA resin/ MB1: IvoBase CAD, Ivoclar Vivodent, MB2: Polident PMMA, Polident Dental/ Specimen/ NR/ Beam/ NR/ PMMA based resin/ Microfiller reinforced polymer matrix composite/ VITA Vitapan XL T44, #8, VITA Zahnfabrik/ NR/ Tooth shape/ Ivobase CAD bond, Ivoclar Vivodent/ Mechanical treatment | 18  (9/9) | Distilled water/ 24/ NA/ Shear bond strength/ Universal Testing Machine/ Instron, Instron Corp./ 0.5 | T1, MB1, L1:  141.86 ± 21.05  T2, MB2, L2:  108 ± 12.98 | MB1:  150.28 ± 17.36  MB2:  180 ± 34.90 | For complete denture wearers, all resin materials used in this study may be clinically acceptable, as the sheer stress for all groups was higher than the reported  maximum biting force of complete denture patients |
| Choi et al./ 2020/ New Zealand/ (Choi et al., 2020) | Digital model/ NA/ NA/ NR/ | DLP /Cara Print 4.0, Kulzer/ non-PMMA resin/ Dima Print Denture Base, Kulzer/ NR/ NR/ Specimen/ 25 x 4 x 3 mm/ Beam/ Polishing/ non-PMMA resin/ multi-constituent substance/ Dima Print Denture Teeth, Kulzer/ 25 x 4 x 3 mm/ Beam/ Dima Print Denture Base, Kulzer/ NR | NR/ NR/ PMMA resin/ IvoBase CAD, Ivoclar Vivodent/ Specimen/ 25 x 4 x 3 mm/ Beam/ Polishing/ PMMA based resin/ CC1: unfilled PMMA, CC2: double cross-linked PMMA, CC3: PMMA with nanofillers/ TB1: Ivoclar SPE, Ivoclar Vivodent, TB2: Ivoclar DCL, Ivoclar Vivodent, TB3: Mondial, Kulzer/ 25 x 4 x 3 mm/ Beam/ Ivobase CAD bond, Ivoclar Vivodent/ NR | 20  (10/10) | AP1: no aging, AP2: Thermocycling/ AD1: NA, AD2: 6, AD3: 12/ TC1: NA, TC2: 600, TC3: 1200/ Shear bond strength/ Universal Testing Machine/ Instron, Instron Corp./ NR | AP1, AD1, TC1:  0.55 ± 0.14  AP2, AD2, TC2:  0.55 ± 0.18  AP2, AD3, TC3:  0.59 ± 0.25 | CC1, TB1, AP1, AD1, TC1:  1.14 ± 0.47  CC2, TB2, AP1, AD1, TC1:  1.22 ± 0.38  CC3, TB3, AP1, AD1, TC1:  2.21 ± 0.86  CC1, TB1, AP2, AD2, TC2:  1.22 ± 0.39  CC2, TB2, AP2, AD2, TC2:  1.22 ± 0.38  CC3, TB3, AP2, AD2, TC2:  1.03 ± 0.32  CC1, TB1, AP2, AD3, TC3:  1.78 ± 0.52  CC2, TB2, AP2, AD3, TC3:  1.43 ± 0.29  CC3, TB3, AP2, AD3, TC3:  1.26 ± 0.27 | The bond strength  decreased significantly with aging. Teeth bonded to CAD-CAM and 3D printed DBRs showed  significantly lower bond strength, with no significant influence of aging. |
| Mohamed et al./ 2023/ Japan/ (A. Mohamed et al., 2023) | Intaglio surface of denture tooth/ Extraoral scanner/ EDGE 3D Model Scanner, DOF Inc./ Meshmixer, Autodesk | DLS/ M2, Carbon/ non-PMMA resin/ Lucitone Digital Print, Dentsply Sirona/ NR/ 90/ Specimen/ 20 x 10 mm/ Cylindrical/ Cured in a specialized light-curing unit (InLab Speedcure Processing Unit, Dentsply Sirona) for 26'/ PMMA based resin/ Interpenetrating polymer network/ IPN 3D print tooth material, Dentsply Sirona/ 9 x 11 mm/ Tooth shape/ Lucitone digital fuse step 2, Dentsply Sirona/ NR | NR/ NR/ PMMA resin/ XCL1; AvaDent, AvaDent Digital Dental Solutions/ Specimen/ 20 x 10 mm/ Cylindrical/ Finishing, Polishing/ PMMA based resin/ Extreme cross-linked PMMA/ XCL1; AvaDent, Digital Dental Solutions/ 9 x 11 mm/ Tooth shape/ NA/ NA | 20  (10/10) | AP1: no aging, AP2: Thermocycling/ AD1: NA, AD2: 12/ TC1: NA, TC2: 10000/ Shear bond strength/ Universal Testing Machine/ AG-X, Shimadzu Corp./ 1 | AP1, AD1, TC1:  5.77 ± 1.71  AP2, AD2, TC2:  5.86 ± 0.71 | AP1, AD1, TC1:  7.76 ± 0.90  AP2, AD2, TC2:  7.18 ± 0.89 | The milled monolithic fabrication technique, which eliminates the need for a bonding step, offered a  promising combination of high-precision digital fabrication and a significantly high BS |
| Alanazi et al./ 2024/ UK/ (Alanazi et al., 2024) | Acrylic removable denture bases/ Intraoral scanner/ NR/ Fusion 360, Autodesk | T1: FDM, T2: SLA/B1: LulzBot TAZ6, Aleph Objects Inc., B2: Form 2, Formlabs/ M1: PMMA resin, M2: non-PMMA resin/ MB1: 3D filament, Material4print, MB2: Grey Resin, Formlabs, MB3: Denture Base Resin, Formlabs/ NR/ BO1: 90, BO2: 0/ Denture base/ 64 × 10 × 3.3 mm/ Denture shape/ Unprocessed/ NR/ CC1: NR, CC2: plastic teeth, CC3: printed teeth/ NR/ Based on ISO specifications/ BA1: NR, BA2: Uncured denture base resin, BA3: Self-cure resin (ortho resin)/ TP1: No treatment, TP2: Mechanical and chemical treatment | 5-axis/ Roland DWX‐50/ PMMA resin/ IvoBase CAD, Ivoclar Vivodent/ Denture base/ 64 × 10 × 3.3 mm/ Denture shape/ Unprocessed/ NR/ NR/ NR/ Based on ISO specifications/ Based on ISO specifications/ NR/ TP1: No treatment, TP2: Mechanical and chemical treatment | 12  (6/6) | NA/ NA/ NA/ Shear bond strength/ Universal Testing Machine/ Lloyd LRX, AMETEK Inc/ 1 | T1, B1, M1, MB1, BO1, CC1, BA1, TP1:  132.52 ± 40.78  T1, B1, M1, MB1, BO2, CC1, BA1, TP1:  111.38 ± 22.88  T2, B2, M2, MB2, BO1, CC1, BA1, TP1:  116.26 ± 32.5  T2, B2, M2, MB2, BO2, CC1, BA1, TP1:  90.24 ± 26.53  T2, B2, M2, MB3, BO1, CC2, BA1, TP1:  140.65 ± 50.75  T2, B2, M2, MB3, BO2, CC2, BA1, TP1:  76.42 ± 40.47  T2, B2, M2, MB3, BO1, CC2, BA1, TP1:  363.27 ± 51.44  T2, B2, M2, MB3, BO2, CC2, BA1, TP1:  297.55 ± 51.44  T2, B2, M2, MB3, BO2, CC1, BA2, TP2:  229.79 ± 58.27  T2, B2, M2, MB3, BO2, CC1, BA3, TP2:  237.3 ± 130.36 | TP1:  96.33 ± 21.57  TP2:  298.66 ± 130.36  TP2:  298.66 ± 73.62 | These constructs do not comply with ISO specifications  for tooth bonding |
| Löscher et al./ 2024/ Germany/ (Löscher et al., 2024) | Digital model/ NA/ NA/ NR | SLA /Form 3B+, Formlabs/ non-PMMA resin/ Denture Base Resin, Formlabs/ 50/ 0/ Specimen/ NR/ Denture shape/ Alcohol cleaning, Postcuring/ TM1: PMMA-based resin, TM2: non-PMMA resin/ CC1: SE polymer composite, CC2: Microfiller reinforced polymer matrix composite, CC3: NR, CC4: Nano-ceramic filled biocompatible material/ TB1: Vionic Vigo, Vita Zahnfabrik, TB2: Vionic Dent Disc, Vita Zahnfabrik, TB3: Denture Teeth Resin, Formlabs/ NR/ Tooth shape/ BA1: Vionic Bond, Vita Zahnfabrik, BA2: Denture Base Resin, Formlabs, BA3: NA/ TP1: Mechanical treatment, TP2: NA | 5-axis/ B1: CORiTEC 350 Pro+, imes-icore, B2: PrograMill PM7, Ivoclar/ PMMA resin/ MB1: Vionic Base Disc, Vita Zahnfabrik, MB2: Ivotion Disc, Ivoclar Vivodent, MB3: Ivotion Base Disc, Ivoclar Vivodent/ Specimen/ NR/ Denture shape/ NR/ PMMA-based resin/ CC1: SE polymer composite, CC2: Microfiller reinforced polymer matrix composite, CC3: NR, CC4: Monochromatic dental ceramic layer (DCL) material/ TB1: Vionic Vigo, Vita Zahnfabrik, TB2: Vionic Dent Disc, Vita Zahnfabrik, TB3: Ivotion Disc, Ivoclar Vivodent, TB4: Ivotion Dent Disc, Ivoclar Vivodent/ BA1: Vionic Bond, Vita Zahnfabrik, BA2: NA, BA3: Ivotion Bond, Ivoclar Vivodent/ TP1: Mechanical treatment, TP2: NA | 16  (8/8) | Thermocycling/ NR/ 6000/ Shear bond strength/ Universal Testing Machine/ Z010, ZwickRoell/ 1 | TM1, CC1, TB1, BA1, TP1:  1.73 ± 1.28  TM1, CC1, TB1, BA2, TP1:  2.10 ± 0.50  TM1, CC2, TB2, BA1, TP1:  3.46 ± NR  TM2, CC3, TB3, BA3, TP2:  6.98 ± 1.41  TM2, CC4, TB3, BA1, TP1:  2.01 ± NR  TM2, CC4, TB3, BA2, TP1:  0.03 ± 0.02 | B1, MB1, CC1, TB1, BA1, TP1:  2.87 ± 0.78  B1, MB1, CC2, TB2, BA1, TP1:  3.78 ± 0.82  B2, MB2, CC3, TB3, BA2, TP2:  10.40 ± 0.73  B2, MB3, CC4, TB4, BA3, TP1:  3.69 ± 0.46 | Milled,  pressed and prefabricated systems provided longer survival  and fracture force than the other tested systems. |

SM: Subtractive Manufacturing, AM: Additive Manufacturing, T: Technology, SLA: Stereolithography Apparatus, DLS: Digital Light Synthesis, MB: Material Brand, L: Layer thickness, NR: Not Reported, NA: Not Applicable, DLP: Digital Light Processing, CC: Chemical Composition of Bonded Teeth, TB: Bonded Teeth Brand, AP: Aging Process, AD: Aging Duration, TC: Thermocycling Cycles, FDM: Fused Deposition Modeling, BA: Bonding agent, TP: Teeth pre-treatment, TM: Bonded teeth material.

**Supplementary Table 3.** Subgroup meta-analysis results for reline–base bond strength comparisons between additive and subtractive denture bases.

| **Author/ Year/Country/ Reference** | **Study model/ digitalization method/ digitalization model and brand/ design software (AM)/ design software (SM)** | **3D-printing technology/ brand/ material/ material brand/ layer thickness (µm)/ build orientation/ specimen type/ specimen dimension/ specimen type/ denture specimen dimension/ liner specimen dimension/ specimen shape/ post-processing approach** | **Milling machine setting/ brand/ material/ material brand/ specimen type/ specimen dimension/ liner specimen dimension/ specimen shape/ post-processing approach** | **Type of liner/ Type of polymerization of liner/ Liner material/ Liner brand/ Surface preparation of denture resin specimens (Before liner usage)/ bonding agent (AM)/ bonding agent (SM)/ Specimen pre-treatment before bond strength test** | **Total sample size (AM/ SM)** | **crosshead speed(mm/min)/ evaluation test/ test machine/ test machine brand** | **Reported values for the studied measurement outcomes (mean ± SD) for AM liner bond strength (MPa)/ failure mode (%): Adhesive, Cohesive, Mixed** | **Reported values for studied measurement outcomes (mean ± SD) for SM liner bond strength (MPa)/ failure mode (%): Adhesive, Cohesive, Mixed** | **Conclusion(s)** |
| --- | --- | --- | --- | --- | --- | --- | --- | --- | --- |
| Alfaraj et al./ 2023/ USA/ (Alfaraj et al., 2023) | Digital model/ NR/ NR | DLS/ M2, Carbon/ non-PMMA resin/ Lucitone Digital Print, Dentsply Sirona/ NR/ NR/ Specimen/ 80 × 7 × 2 mm/ 3 x 7 x 2 mm/ Beam/ Polishing | 5-axis/ RolandDWX-51D/ PMMA-based resin/ IvoBase CAD, Ivoclar Vivadent/ Specimen/ 80 × 7 × 2 mm/ 3 x 7 x 2 mm/ Beam/ Polishing | Hard/ Auto/ Acrylate-based/ LB1: ProBase Cold, Ivoclar Vivadent, LB2: Lucitone 199 denture base material, Dentsply Sirona/ Mechanical treatment/ Bonding adhesive/ Methacrylate monomer/ Polishing, storing in distilled water for 7 days | 20  (10/10) | 5/ Tensile bond strength/ Universal Testing Machine/ MTS Sintech ReNew, MTS Systems Corp | LB1:  7.2 ± 0.9/ NR, NR, NR  LB2:  12.7 ± 4.5/ NR, NR, NR | LB1:  13.3 ± 1.7/ NR, NR, NR  LB2:  18.8 ± 3.2/ NR, NR, NR | Although the heat-polymerizing reliner led to a higher TBS than the autopolymerizing  reliner in most denture base materials, the compression-molding denture  base material can achieve high TBS with both reliners. |
| Vuksic et al./ 2023/ Croatia/ (Vuksic et al., 2023) | Digital model/ NR/ NR | DLP/ NR/ M1: non-PMMA resin, M2: PMMA-based resin/ MB1: Freeprint denture, Detax, MB2: Imprimo LC denture, Scheu/ NR/ NR/ Specimen/ 25 x 25 x 3 mm/ 10 x 3 mm/ Plate/Block + Cylindrical/ Water cleaning, Polishing | NR/ NR/ PMMA-based resin/ MB1: IvoBase CAD, Ivoclar Vivadent, MB2: Polident, Polident d.o.o., MB3: Anaxdent, Anaxdent/ Specimen/ 25 x 25 x 3 mm/ 10 x 3 mm/ Plate/Block + Cylindrical/ Polishing | Soft/ Auto/ LM1: Acrylate-based, LM2: Silicone-based/ LB1: GC Soft liner, GC Corp., LB2: Reline II soft, GC Corp./ No treatment/ Bonding adhesive/ Bonding adhesive/ Storing in distilled water for 23 h | 20  (10/10) | 10/ Tensile bond strength/ Universal Testing Machine/ AutographAGS-X, Shimadzu | M1, MB1, LM1:  0.20 ± 0.07/ 70, 30, 10  M1, MB1, LM2:  1.89 ± 0.47/ 0, 50, 50  M2, MB2, LM1:  0.19 ± 0.09/ 90, 10, 0  M2, MB2, LM2:  1.80 ± 0.50/ 0, 80, 20 | MB1, LM1:  0.24 ± 0.08/ 0, 90, 10  MB1, LM2:  2.85 ± 0.23/ 80, 20, 0  MB2, LM1:  0.25 ± 0.08/ 0, 100, 0  MB2, LM2:  3.07 ± 0.23/ 30, 60, 10  MB3, LM1:  0.24 ± 0.08/ 0, 100, 0  MB3, LM2:  2.74 ± 0.27/ 60, 40, 0 | Significant differences in  tensile bond strength values were found between the silicone-based soft denture liner and denture  base materials, where the additive-manufactured and polyamide denture base materials showed  lower values than heat-cured PMMA and subtractive-manufactured denture base materials. |
| Wemken et al./ 2021/ Germany/ (Wemken et al., 2021) | Digital model/ Fusion360, Autodesk/ Fusion360, Autodesk | DLP/ SolFlex 170, VOCO/ non-PMMA resin/ V-Print dentbase, VOCO/ 50/ 0/ Specimen/ 10 × 10 × 43 mm/ 10 × 10 × 3 mm/ Beam/ Alcohol cleaning, Post-curing, Polishing | 5-axis/ Zenotec Select, WielandDental/ PMMA-based resin/ IvoBase CAD, Ivoclar Vivadent/ Specimen/ 10 × 10 × 43 mm/ 10 × 10 × 3 mm/ Beam/ Polishing | L1: soft, L2: hard/ Auto/ LM1: Silicone-based, LM2: Acrylate-based/ LB1: Ufi Gel SC, VOCO, LB2: Ufi Gel hard C, VOCO. LB3: PalaXpress, Kulzer/ No treatment/ BA1: Bonding adhesive, BA2: Methacrylate monomer/ BA'1: Bonding adhesive, BA'2: Methacrylate monomer/ SP1: Storing in distilled water for 50 h, SP2: Hydrothermally cycled in water set at 5◦C and 55◦C with 30' each for 5000 cycles, SP3: Storing in 350ml of distilled water while being exposed to microwaves (640W) for 6 cycles of 3" at intervals of 1 h | 16  (8/8) | 5/ Tensile bond strength/ Universal Testing Machine/ Z010, ZwickRoell | L1, LM1, LB1, BA1, SP1:  1.2 ± 0.1/  100, 0, 0  L1, LM1, LB1, BA1, SP2:  1.2 ± 0.3/  100, 0, 0  L1, LM1, LB1, BA1, SP3:  1.4 ± 0.3/ 100, 0, 0  L2, LM2, LB2, BA1, SP1:  14.1 ± 3.1/  12.5, 62.5, 25  L2, LM2, LB2, BA1, SP2:  10.7 ± 1.4/ 12.5, 50, 37.5  L2, LM2, LB2, BA1, SP3:  10.9 ± 4.1/  50, 37.5, 12.5  L2, LM2, LB2, BA2, SP1:  11.8 ± 1.6/ 25, 75, 0  L2, LM2, LB2, BA2, SP2:  10.4 ± 3.5/ 25, 75, 0  L2, LM2, LB2, BA2, SP3:  11.4 ± 2.5/ 62.5, 25, 12.5 | L1, LM1, LB1, BA'1, SP1:  1.3 ± 0.2/ 100, 0, 0  L1, LM1, LB1, BA'1, SP2:  1.2 ± 0.3/ 100, 0, 0  L1, LM1, LB1, BA'1, SP3:  1.2 ± 0.2/ 100, 0, 0  L2, LM2, LB2, BA'1, SP1:  5.8 ± 1.7/ 100, 0, 0  L2, LM2, LB2, BA'1, SP2:  5.5 ± 0.9/ 100, 0, 0  L2, LM2, LB2, BA'1, SP3:  5.7 ± 1.9/ 100, 0, 0  L2, LM2, LB2, BA'2, SP1:  21.7 ± 3.2/ 87.5, 12.5, 0  L2, LM2, LB2, BA'2, SP2:  23.7 ± 6.7/ 100, 0, 0  L2, LM2, LB2, BA'2, SP3:  17.5 ± 5.7/ 100, 0, 0 | Bond strength to soft liner was affected neither by the base material nor by the pre-treatment. Hard liner demonstrated twice the bond strength to additive compared with subtractive. |
| Gad et al./ 2022/ Saudi Arabia/ (Gad et al., 2022) | Digital model/ NR/ NR | T1: DLP, T2: SLA/ B1: Asiga Max, SCHEU-DENTAL, B2: NextDent 5100, B3: Form 2, Formlabs/ non-PMMA resin/ MB1: DentaBASE, ASIGA, MB2: Denture 3D+, NextDent, MB3: Denture Base LP Resin, Formlabs/ 50/ 0/ Specimen/ 10 x 10 x 3 mm/ 4 x 6 mm/ Plate/Block + Cylindrical/ Polishing | NR/ NR/ PMMA-based resin/ MB1: IvoBase CAD, Ivoclar Vivadent, MB2: AvaDent, AvaDent Digital Dental Solutions/ Specimen/ 10 x 10 x 3 mm/ 4 x 6 mm/ Plate/Block + Cylindrical/ Polishing | Hard/ Auto/ Acrylate-based/ Major repair, Major Prodotti Dentari SPA/ SPD1: No treatment, SPD2: Chemical treatment, SPD3: Mechanical treatment/ BA1: No bonding agent, BA2: Methacrylate monomer/ BA'1: No bonding agent, BA'2: Methacrylate monomer/ Storing in distilled water for 48 h, Thermocycling for 5000 cycles at 5°C to 55°C with a 30' dwell time | 20  (10/10) | 0.5/ Shear bond strength/ Universal Testing Machine/ Instron, Instron Corp. | T1, B1, MB1, SPD1, BA1:  2.77 ± 0.35/ 100, 0, 0  T1, B1, MB1, SPD2, BA2:  3.47 ± 0.99/ 80, 10, 10  T1, B1, MB1, SPD3, BA1:  5.81 ± 0.88/ 30, 50, 20  T1, B2, MB2, SPD1, BA1:  1.91 ± 0.23/ 100, 0, 0  T1, B2, MB2, SPD2, BA2:  2.23 ± 0.35/ 70, 10, 20  T1, B2, MB2, SPD3, BA1:  5.85 ± 0.97/ 20, 50, 30  T2, B3, MB3, SPD1, BA1:  2.26 ± 0.32/ 90, 0, 10  T2, B3, MB3, SPD2, BA2:  3.81 ± 0.72/ 60, 0, 40  T2, B3, MB3, SPD3, BA1:  6.46 ± 0.92/ 30, 50, 20 | MB1, SPD1, BA'1:  6.87 ± 0.89/ 60, 10, 30  MB1, SPD2, BA'2:  12.19 ± 1.39/ 30, 10, 60  MB1, SPD3, BA'1:  16.14 ± 0.95/ 0, 30, 70  MB2, SPD1, BA'1:  7.08 ± 1.04/ 40, 10, 50  MB2, SPD2, BA'2:  11.84 ± 1.05/ 20, 20, 60  MB2, SPD3, BA'1:  16.64 ± 1.58/ 0, 40, 60 | All materials with mechanical treatment showed a significant  increase in soft liner bond strength when compared with the controls and chemical treatment. |
| Awad et al./ 2023/ USA/ (Awad et al., 2023) | Digital model/ Meshmixer, Autodesk/ Meshmixer, Autodesk | SLA/ NR/ non-PMMA resin/ Denture Base LP Resin, Formlabs/ NR/ 0/ Specimen/ 10 x 10 × 20 mm/ 10 × 10 × 3 mm/ Beam/ Cleaning | NR/ NR/ PMMA-based resin/ IvoBase CAD, Ivoclar Vivadent/ 10 x 10 × 20 mm/ 10 × 10 × 3 mm/ Beam/ NR | L1: Soft, L2: Hard/ Auto/ Acrylate-based/ LB1: COE Soft, GC Corp., LB2: PermaSoft, Dentsply Sirona, LB3: Tokuyama Rebase II, Tokuyama Dental Corp., LB4: Kooliner, GC America, LB5: ProBase Cold, Ivoclar Vivadent/ No specific surface treatment/ NR/ NR/ Storing in distilled water for 24 h | 20  (10/10) | 5/ Tensile bond strength/ Universal Testing Machine/ Instron, Instron Corp. | L1, LB1:  0.2 ± 0.0/ NR, NR, NR  L1, LB2:  0.4 ± 0.0/ NR, NR, NR  L2, LB3:  4.2 ± 0.7/ NR, NR, NR  L2, LB4:  5.5 ± 0.8/ NR, NR, NR  L2, LB5:  14.7 ± 1.3/ NR, NR, NR | L1, LB1:  0.2 ± 0.0/ NR, NR, NR  L1, LB2:  0.5 ± 0.0/ NR, NR, NR  L2, LB3:  6.0 ± 1.5/ NR, NR, NR  L2, LB4:  6.4 ± 1.2/ NR, NR, NR  L2, LB5:  16.4 ± 1.9/ NR, NR, NR | The printed denture bases had significantly lower tensile bond strength values than the injection and milled denture bases with the PermaSoft, Tokuyama Rebase ii, and ProBase Cold denture relines, while milled denture bases demonstrated the highest values of  tensile bond strength for all chairside relining groups. |
| Gibreel et al./ 2024/ Finland/ (Gibreel et al., 2024) | Digital model/ Fusion 360, Autodesk/ Fusion 360, Autodesk | DLP/ Asiga Max, SCHEU-DENTAL/ Non-PMMA resin/ MB1: V-Print dentbase, VOCO, MB2: Freeprint denture, Detax/ 50/ NR/ Specimen/ 20 × 10 × 3 mm/ 3.6 x 3 mm/ Plate-Block + Cylindrical/ Alcohol cleaning, Post-curing, Polishing | NR/ NR/ PMMA-based resin/ MB1: L-Temp, Degos Dental, MB2: Temp Basic Tissue, Zirkonzahn/ Specimen/ 20 × 10 × 3 mm/ 3.6 x 3 mm/ Plate/Block + Cylindrical/ Polishing | Hard/ Auto/ Acrylate-based/ Palapress, Kulzer/ SPD1: No treatment, SPD2: Chemical treatment, SPD3: Mechanical and chemical treatment, SPD4: Mechanical treatment/ BA1: No bonding agent, BA2: Methacrylate monomer/ BA'1: No bonding agent, BA'2: Methacrylate monomer/ Storing in distilled water at 100 °C for 16 h | 14  (7/7) | 1/ Shear bond strength/ Universal Testing Machine/ Lloyd LRX, AMETEK Inc. | MB1, SPD1, BA1:  7.5 ± 3.3/  57, 43, 0  MB1, SPD2, BA2:  10.9 ± 3.1/  14, 86, 0  MB1, SP2, BA1:  9.1 ± 2.5/  29, 43, 29  MB1, SPD3, BA2:  12.1 ± 3.3/  0, 100, 0  MB1, SPD4, BA1:  15.0 ± 5.8/  0, 100, 0  MB2, SPD1, BA1:  9.9 ± 2.9/  0, 100, 0  MB2, SPD2, BA2:  9.3 ± 3.4/  0, 100, 0  MB2, SPD2, BA1:  7.8 ± 1.4/  0, 100, 0  MB2, SPD3, BA2:  19.3 ± 5.8/  0, 100, 0  MB2, SPD4, BA1:  17.3 ± 3.0/  0, 100, 0 | MB1, SPD1, BA'1:  17.7 ± 5.2/  14, 57, 29  MB1, SPD2, BA'2:  23.3 ± 3.9/  0, 100, 0  MB1, SP2, BA'1:  19.2 ± 5.3/  14, 57, 29  MB1, SPD3, BA'2:  21.3 ± 3.4/  14, 86, 0  MB1, SPD4, BA'1:  19.5 ± 5.8/  0, 86, 14  MB2, SPD1, BA'1:  26.6 ± 5.6/  0, 100, 0  MB2, SPD2, BA'2:  28.8 ± 5.3/  0, 100, 0  MB2, SPD2, BA'1:  27.7 ± 3.6/  0, 100, 0  MB2, SPD3, BA'2:  24.3 ± 4.4/  0, 100, 0  MB2, SPD4, BA'1:  24.9 ± 6.9/  0,100, 0 | The bond strength of CAD-CAM-milled denture base resins to autopolymerizing repair resins is greater than 3d printed ones. Surface roughening using air particle abrasion or 180-grit carbide paper can enhance the  bond strength of the autopolymerizing repair resin to 3D-printed denture base materials. |
| Sahin et al./ 2024/ Turkey/ (Sahin et al., 2024b) | Digital model/ SolidWorks, Dassault Systemes/ SolidWorks, Dassault Systemes | LCD/ FreeShape 120/ non-PMMA resin/ Curo Denture, MACK4D, Ackuretta Technologies Pvt Ltd./ 50/ 0/ Specimen/ 7.5 x 2 mm/ 3 x 5 mm/ Cylindrical/ Dimensionally checking, Embedded in PMMA auto polymerized acrylic resin, Polishing, storing in distilled water for 24 h, Thermal cycling (5000 cycles, ranging from 5 to 55 °C) | 5-axis/ CORiTEC 250i, imes-icore/ PMMA-based resin/ Yamahachi, Yamahachi Dental MFG/ Specimen/ 7.5 x 2 mm/ 3 x 5 mm/ Cylindrical/ Dimensionally checking, Embedded in PMMA auto polymerized acrylic resin, Polishing, storing in distilled water for 24 h, Thermal cycling (5000 cycles, ranging from 5 to 55 °C) | Hard/ Auto/ Acrylate-based/ Meliodent, Kulzer/ SPD1: No treatment, SPD2: Mechanical treatment, SPD3: Mechanical treatment, SPD4: Physiochemical treatment, SPD5: Physiochemical treatment / No bonding agent/ No bonding agent/ Thermocycling for 5000 cycles at 5°C to 55°C | 20  (10/10) | 1/ Shear bond strength/ Universal Testing Machine/ Lloyd LRX, AMETEK Inc | SPD1:  11.84 ± 8.49/ 100, 0, 0  SPD2:  11.5 ± 6.21/ 100, 0, 0  SPD3:  27.2 ± 13.58/ 60, 40, 0  SPD4:  32.88 ± 12.72/  80, 20, 0  SPD5:  5.66 ± 3.86/ 100, 0, 0 | SPD1:  28.2 ± 18.8/ 100, 0, 0  SPD2:  55.81 ± 26.93/  100, 0, 0  SPD3:  56.96 ± 21.31/  70, 30, 0  SPD4:  44.39 ± 19.11/  90, 10, 0  SPD5:  21.94 ± 12.47/  100, 0, 0 | laser treatment was the most effective in the additive-manufactured group. For the subtractive group, surface treatments other than plasma exhibited similar bond strength. |
| Karaokutan et al./ 2024/ Turkey/ (Karaokutan and Ayvaz, 2025) | Digital model/ Meshmixer, Autodesk/ Meshmixer, Autodesk | DLP/ SolFlex 650, VOCO/ non-PMMA resin/ V-Print dentbase, VOCO/ 65/ 90/ Specimen/ 10 x 2 mm/ 3 x 5 mm/ Cylindrical/ Alcohol Cleaning, Post-curing, Polishing | 5-axis/ CORiTEC 550i, imes-icore/ PMMA-based resin/ Polident, Pearson Dental Supply/ Specimen/ 10 x 2 mm/ 3 x 5 mm/ Cylindrical/ Polishing | Hard/ Auto/ Acrylate-based/ Paladur, Kulzer/ SPD1: Chemical treatment, SPD2: Mechanical treatment, SPD3: Mechanical treatment, SPD4: Mechanical treatment/ BA1: Methacrylate monomer, BA2: No bonding agent/ BA'1: Methacrylate monomer, BA'2: No bonding agent/ Storing in distilled water for 24 h, Thermocycling for 5000 cycles at 5°C to 55°C with a 20' dwell time | 20  (10/10) | 1/ Shear bond strength/ Universal Testing Machine/ NR | SPD1:  1.99 ± 0.61/ 100, 0, 0  SPD2:  19.46 ± 0.92/ 50, 20, 30  SPD3:  3.89 ± 0.56/ 100, 0, 0  SPD4:  7.98 ± 0.64/ 80, 0, 20 | SPD1:  3.67 ± 0.49/ 100, 0, 0  SPD2:  16.18 ± 0.59/ 70, 10, 20  SPD3:  9.27 ± 0.54/ 80, 0, 20  SPD4:  8.2 ± 0.59/ 80, 0, 20 | Mechanical surface treatments and universal adhesive applications are  more effective for maintaining adhesion across all production techniques. |
| Mert et al./ 2023/ Switzerland/ (Mert et al., 2023) | Digital model/ NR/ NR | NR/ NR/ non-PMMA resin/ Denture 3D+, NextDent/ NR/ NR/ Specimen/ 10 × 10 × 11 mm/ 10 x 10 x 3 mm/ Beam/ Finishing, Polishing | NR/ NR/ PMMA-based resin/ MB1: IvoBase CAD, Ivoclar Vivadent, MB2: Ivotion, Ivoclar Vivadent/ Specimen/ 10 × 10 × 11 mm/ 10 x 10 x 3 mm/ Beam/ Finishing, Polishing | Hard/ Auto/ Acrylate-based/ ProBase Cold, Ivoclar Vivadent/ No treatment/ Methacrylate monomer/ Methacrylate monomer/ Thermocycling for 10,000 cycles at 5°C to 55°C/ | 24  (12/12) | 1/ Shear bond strength/ Universal Testing Machine/ Z010, ZwickRoell | 25.897 ±  6.758/  0, 0, 100 | MB1:  36.575 ± 5.030/  0, 0, 100  MB2:  40.315 ± 6.512/  0, 0, 100 | The findings of this study conclude that the shear bond strength of the conventionally-relined 3D-printed resins used for fabricating CRDPs was inferior to the shear bond strength of conventionally-relined resins employed for manufacturing CRDPs using CAD-CAM milling. |
| Albazroun et al./ 2024/ Saudi Arabia/ (Albazroun et al., 2024) | Digital model/ NR/ NR | DLP/ P1: NextDent 5100, NextDent, P2: Form2, Formlabs/ non-PMMA resin/ MB1: Denture 3D+, NextDent, MB2: Denture Base LP Resin, Formlabs/ 50/ 0/ Specimen/ 10 × 10 × 2.5 mm/ 4 x 6 mm/ Plate/Block + Cylindrical/ Polishing | NR/ NR/ PMMA-based resin/ MB1: IvoBase CAD, Ivoclar Vivadent, MB2: AvaDent, AvaDent Digital Dental Solutions/ Specimen/ 10 × 10 × 2.5 mm/ 4 x 6 mm/ Plate/Block + Cylindrical/ Polishing | Hard/ Auto/ Acrylate-based/ GC RELINE, GC Corp./ No treatment/ No bonding agent/ No bonding agent/ Storing in distilled water for 48 h, Thermocycling for 5000 cycles at 5°C to 55°C with a 30' dwell time | 20  (10/10) | 1/ Shear bond strength/ Universal Testing Machine/ Instron, Instron Corp. | P1, MB1:  31.1 ± 11.9/  70, 10, 20  P2, MB2:  29.7 ± 9.2/  80, 20, 0 | MB1:  43.2 ± 6.9/  80, 0, 20  MB2:  51.8 ± 13.3/  80, 0, 20 | CAD-CAM milled denture base resin showed higher bond strength with pure denture reline. |
| Karaokutan et al./ 2024/ Turkey/ (Karaokutan et al., 2024) | Digital model/ Meshmixer, Autodesk/ Meshmixer, Autodesk | DLP/ SolFlex 650, VOCO/ non-PMMA resin/ V-Print dentbase, VOCO/ 65/ 90/ Specimen/ 10 x 2 mm/ 3 x 5 mm/ Cylindrical/ Alcohol cleaning, Postcuring, Polishing | 5-axis/ CORiTEC 550i, imes-icore/ PMMA-based resin/ Polident, Pearson Dental Supply/ Specimen/ 10 x 2 mm/ 3 x 5 mm/ Cylindrical/ Polishing | Hard/ Auto/ Acrylate-based/ Ufi Gel hard C, VOCO/ SPD1: No treatment, SPD2: Mechanical treatment, SPD3: Mechanical treatment, SPD4: Chemical treatment, SPD5: Mechanical and chemical treatment/ BA1: No bonding agent, BA2: Bonding adhesive/ BA'1: No bonding agent, BA'2: Bonding adhesive/ SP1: Storing in distilled water for 24 h, Thermocycling for 5000 cycles at 5°C to 55°C with a 20' dwell time, SP2: Tetrahydrofuran, SP3: Storing in distilled water for 24 h | 20  (10/10) | 1/ Shear bond strength/ Universal Testing Machine/ NR | SPD1, SP1, BA1:  5.95 ± 0.63/ 100, 0, 0  SPD2, SP1, BA1:  12.33 ± 4.81/  70, 0, 30  SPD3, SP1, BA1:  15.51 ± 0.32/  80, 0, 20  SPD4, SP1, BA2:  15.14 ± 0.32/  80, 0, 20  SPD5, SP2, BA2:  8.45 ± 5.15/  100, 0, 0  SPD1, SP3, BA1:  18.06 ± 0.76/  100, 0, 0  SPD2, SP3, BA1:  35.77 ± 0.6/  0, 70, 30  SPD3, SP3, BA1:  36.05 ± 0.41/  80, 0, 20  SPD4, SP3, BA2:  39.23 ± 0.51/  0, 80, 20  SPD5, SP3, BA2:  16.99 ± 0.44/  100, 0, 0 | SPD1, SP1, BA'1:  11.15 ± 1.49/  100, 0, 0  SPD2, SP1, BA'1:  15.79 ± 3.51/  70, 0, 30  SPD3, SP1, BA'1:  16.60 ± 0.89/  100, 0, 0  SPD4, SP1, BA'2:  14.06 ± 1.3/  100, 0, 0  SPD5, SP2, BA'2:  10.97 ± 0.95/  100, 0, 0  SPD1, SP3, BA'1:  20.71 ± 0.76/  100, 0, 0  SPD2, SP3, BA'1:  36.66 ± 0.28/  0, 60, 40  SPD3, SP3, BA'1:  36.55 ± 0.38/  80, 0, 20  SPD4, SP3, BA'2:  32.81 ± 0.63/  10, 10, 80  SPD5, SP3, BA'2:  18.65 ± 0.32/  100, 0, 0 | Among the surface treatment methods, treating denture bases with 50  *μ*m airborne-particle abrasion is more effective for maintaining adhesion, especially in  the additive technique. |
| Htat et al./ 2024/ Thailand/ (Htat et al., 2024) | Digital model/ 3Shape Dental System DS, 3Shape/ 3Shape Dental System DS, 3Shape | DLP/ NextDent 5100, NextDent/ non-PMMA resin/ Denture 3D+, NextDent/ 50/ 90/ Specimen/ 8 x 8 x 8 mm/ 4 x 3 mm/ Plate/Block + Cylindrical/ Alcohol cleaning, Postcuring, Polishing | 5-axis/ DWX-52D, DGShape/ PMMA-based resin/ Smile Cam, Pressing Dental Srl/ Specimen/ 8 x 8 x 8 mm/ 4 x 3 mm/ Plate/Block + Cylindrical/ Polishing | Hard/ Auto/ Acrylate-based/ Unifast Trad, GC Corp./ SPD1: No treatment, SPD2: Mechanical treatment, SPD3: Chemical treatment, SPD4: Chemical treatment/ BA1: No bonding agent, BA2: Bonding adhesive/ BA'1: No bonding agent, BA'2: Bonding adhesive/ Thermocycling for 5000 cycles at 5°C to 55°C with a 30' dwell time | 20  (10/10) | 1/ Shear bond strength/ Universal Testing Machine/ EZ-S, Shimadzu | SPD1, BA1:  16.48 ± 1.58/ 0, 80, 20  SPD2, BA1:  22.52 ± 2.06/  0, 100, 0  SPD3, BA1:  21.08 ± 2.81/  0, 80, 20  SPD4, BA2:  18.05 ± 2.88/ 0, 70, 30 | SPD1, BA'1:  13.52 ± 2.47/  90, 0, 10  SPD2, BA'1:  21.14 ± 1.1/  0, 100, 0  SPD3, BA'1:  14.28 ± 2.68/  90, 0, 10  SPD4, BA'2:  15.45 ± 2.2/  80, 0, 20 | mechanical surface treatment using APA enhances the adhesion of autopolymerizing acrylic resin to CAD-CAM denture bases. Chemical surface treatment with only tetrahydrofuran enhances the bond strength of 3D-printed denture to the level of APA. Without surface treatment, the highest bond strength was shown in 3D-printed denture base material. |
| Janyaprasert et al./ 2024/ Thailand/ (Janyaprasert et al., 2024) | Digital model/ SolidWorks, Dassault Systemes/ SolidWorks, Dassault Systemes | DLP/ NextDent 5100, NextDent/ non-PMMA resin/ Denture 3D+, NextDent/ NR/ NR/ Specimen/ 25 x 25 x 3 mm/ 10 x 3 mm/Plate/ Block + Cylindrical/ Alcohol cleaning, Postcuring, Polishing | 5-axis/ S2 Milling machine, VHF/ PMMA-based resin/ Smile Cam, Pressing Dental/ Specimen/ 25 x 25 x 3 mm/ 10 x 3 mm/Plate/ Block + Cylindrical/ Polishing | L1: Soft, L2: Hard/ Auto/ Acrylate-based/ LB1: GC Soft Liner, GC Corp., LB2: Tokuyama Rebase II, Tokuyama Dental Corp., LB3: Ufi Gel P, VOCO, LB4: Sofreliner Tough M, Tokuyama Dental Corp./ No treatment/ No bonding agent/ No bonding agent/ SP1: Storing in distilled water for 24 h, SP2: Storing in distilled water for 24 h, Thermocycling for 5000 cycles at 5°C to 55°C with a 30' dwell time | 16  (8/8) | 10/ Tensile bond strength/ Universal Testing Machine/ EZ-S, Shimadzu | L1, LB1, SP1:  0.38 ± 0.04/ 100, 0, 0  L1, LB1, SP2:  0.37 ± 0.03/ 62.5, 37.5, 0  L2, LB2, SP1:  0.67 ± 0.13/ 100, 0, 0  L2, LB2, SP2:  0.64 ± 0.25/ 100, 0, 0  L1, LB3, SP1:  1.62 ± 0.23/ 100, 0, 0  L1, LB3, SP2:  0.64 ± 0.25/ 100, 0, 0  L1, LB4, SP1:  1.99 ± 0.48/ 100, 0, 0  L1, LB4, SP2:  2.75 ± 0.59/ 62.5, 12.5, 25 | L1, LB1, SP1:  0.40 ± 0.04/ 0, 100, 0  L1, LB1, SP2:  0.41 ± 0.09/ 0, 100, 0  L2, LB2, SP1:  1.44 ± 0.40/ 100, 0, 0  L2, LB2, SP2:  1.21 ± 0.31/ 100, 0, 0  L1, LB3, SP1:  1.59 ± 0.24/ 100, 0, 0  L1, LB3, SP2:  1.21 ± 0.31/ 100, 0, 0  L1, LB4, SP1:  2.10 ± 0.66/ 87.5, 0, 12.5  L1, LB4, SP2:  2.87 ± 0.43/ 100, 0, 0 | For the non-thermocycling groups, within the same denture liner material, no significant differences were found between denture base materials, except the ND + RB group, which had significantly lower TBS. |
| Tugut et al./ 2024/ Turkey/ (Tugut et al., 2024) | Digital model/ AutoCAD, Autodesk/ SolidWorks, Dassault Systemes | DLP/ Ackuretta/ non-PMMA resin/ MACK4D Denture Light Pink, Dentona/ 100/ 0/ Specimen/ 36 x 7 mm/ 7 × 3 mm/ Cylindrical/ Alcohol cleaning, Postcuring, Polishing | NR/ Redon/ PMMA-based resin/ Yamahachi, Yamahachi Dental MFG/ Specimen/ 36 x 7 mm/ 7 × 3 mm/ Cylindrical/ Polishing | Soft/ Heat/ Silicon-based/ Molloplast-B, Detax/ No treatment/ Bonding adhesive/ Bonding adhesive/ SP1: Storing in distilled water for 48 h, SP2: Storing in distilled water for 48 h, Thermocycling for 5000 cycles at 5°C to 55°C with a 30' dwell time | 20  (10/10) | 5/ Tensile bond strength/ Universal Testing Machine/ Lloyd LF Plus, AMETEK Inc | SP1:  1.36 ± 0.18/ 70, 0, 30  SP2:  1.0 ± 0.14/ 100, 0, 0 | SP1:  1.56 ± 0.62/  60, 0, 40  SP2:  1.36 ± 0.16/ 50, 0, 50 | The bond strength of soft relining materials to CAD/  CAM milled and 3D printed denture base is different. In denture base materials that are CAD/CAM and  3D printed, the thermocycling method reduced bonding strength values. |
| Li et al./ 2024/ USA/ (Li et al., 2025) | Digital model/ Meshmixer, Autodesk | T1: DLP, T2: DLS, T3: SLA/ B1: CARES P30, Straumman, B2: M2, Carbon/ non-PMMA resin/ MB1: FotoDent, Dreve Dentamid, MB2: Dentca, DENTCA, MB3: Lucitone Digital Print, Dentsply Sirona/ NR/ Specimen/ 10 × 10 × 40 mm/ 10 × 10 × 3 mm/ Beam/ Alcohol cleaning, Postcuring, Polishing | 5-axis/ PrograMill PM7, Ivoclar/ PMMA-based resin/ MB1: Luciton 199 Denture Base Disc, Dentsply Sirona, MB2: IvoBase CAD, Ivoclar Vivadent/ Specimen/ 10 × 10 × 40 mm/ 10 × 10 × 3 mm/ Beam/ Polishing | Soft/ Auto/ Acrylate-based/ LB1: COE Soft, GC Corp., LB2: Lynal, Dentsply Sirona/ No specific surface treatment/ No bonding agent/ No bonding agent/ Storing in distilled water for 24 h | 24  (12/12) | 5/ Tensile bond strength/ Universal Testing Machine/ Instron, Instron Corp. | T1, B1, MB1, LB1:  0.12 ± 0.02/ 0, 100, 0  T2, B2, MB2, LB1:  0.16 ± 0.01/ 0, 100, 0  T3, B2, MB3, LB1:  0.19 ± 0.03/ 0, 100, 0  T1, B1, MB1, LB2:  0.09 ± 0.02/ 100, 0, 0  T2, B2, MB2, LB2:  0.15 ± 0.04/ 100, 0, 0  T3, B2, MB3, LB2:  0.22 ± 0.05/ 100, 0, 0 | MB1, LB1:  0.21 ± 0.06/ 0, 100, 0  MB2, LB1:  0.23 ± 0.04/ 0, 100, 0  MB1, LB2:  0.44 ± 0.03/ 91.7, 0, 8.3  MB2, LB2:  0.5 ± 0.06/ 50, 0, 50 | The tensile bond strength between soft relining materials and denture  bases is material-dependent, influenced by both the type of denture base material and  the type of soft relining material used. |

AM: Additive Manufactured, SM: Subtractive Manufactured, NR: Not Reported, DLS: Digital Light Synthesis, LB: Liner Brand, DLP: Digital Light Processing, M: Printing Material, MB: Material Brand, LM: Liner Material, TL: Type of Liner, BA: Bonding Agent (AM), BA': Bonding Agent (SM), SP: Specimen Pre-treatment, SPD: Surface Preparation of Denture, LCD: Liquid Crystal Display.


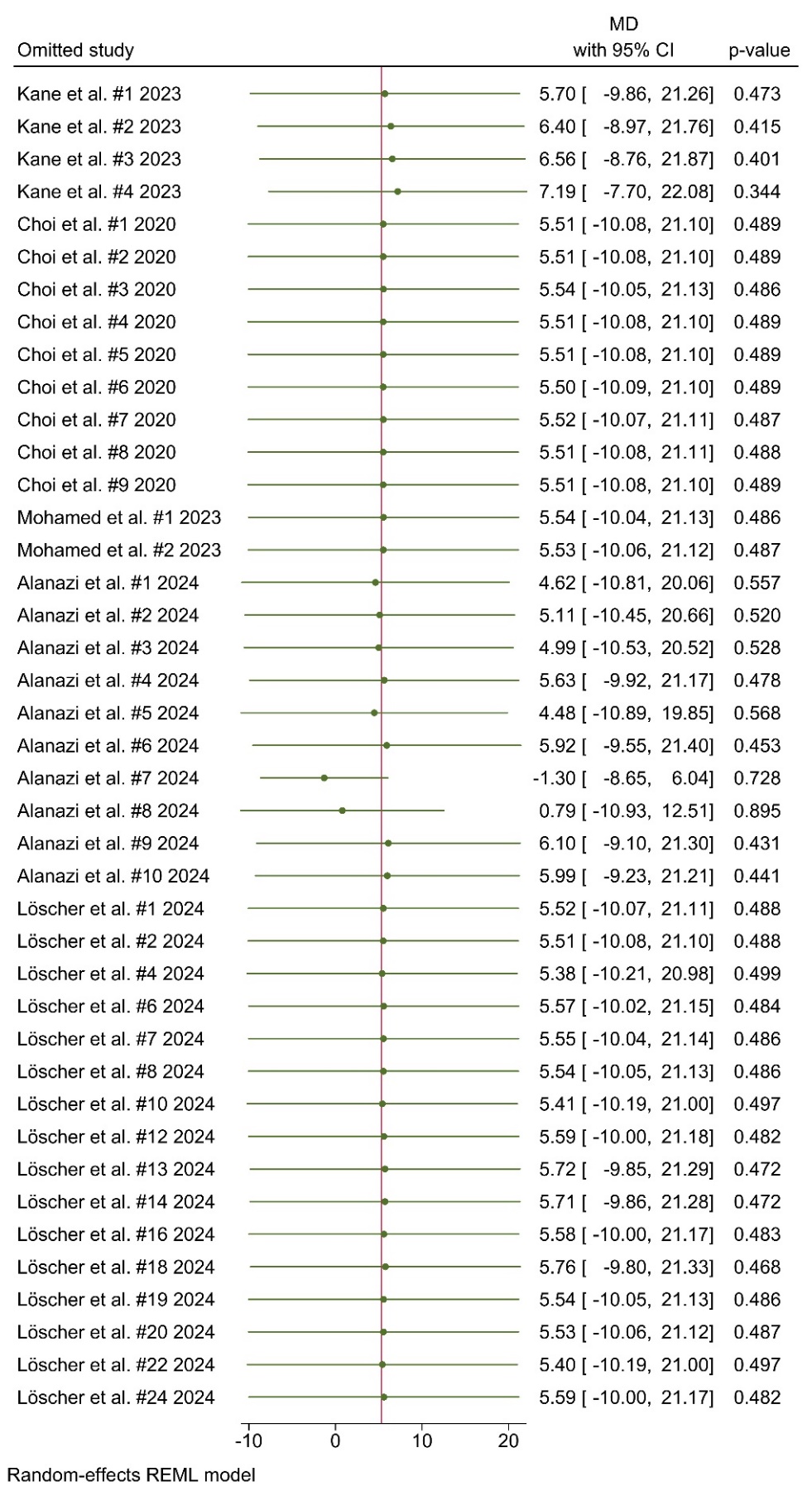


**Supplementary Figure 1.** Leave-one-out sensitivity analysis for tooth–base bonding comparisons before removing the outliers.


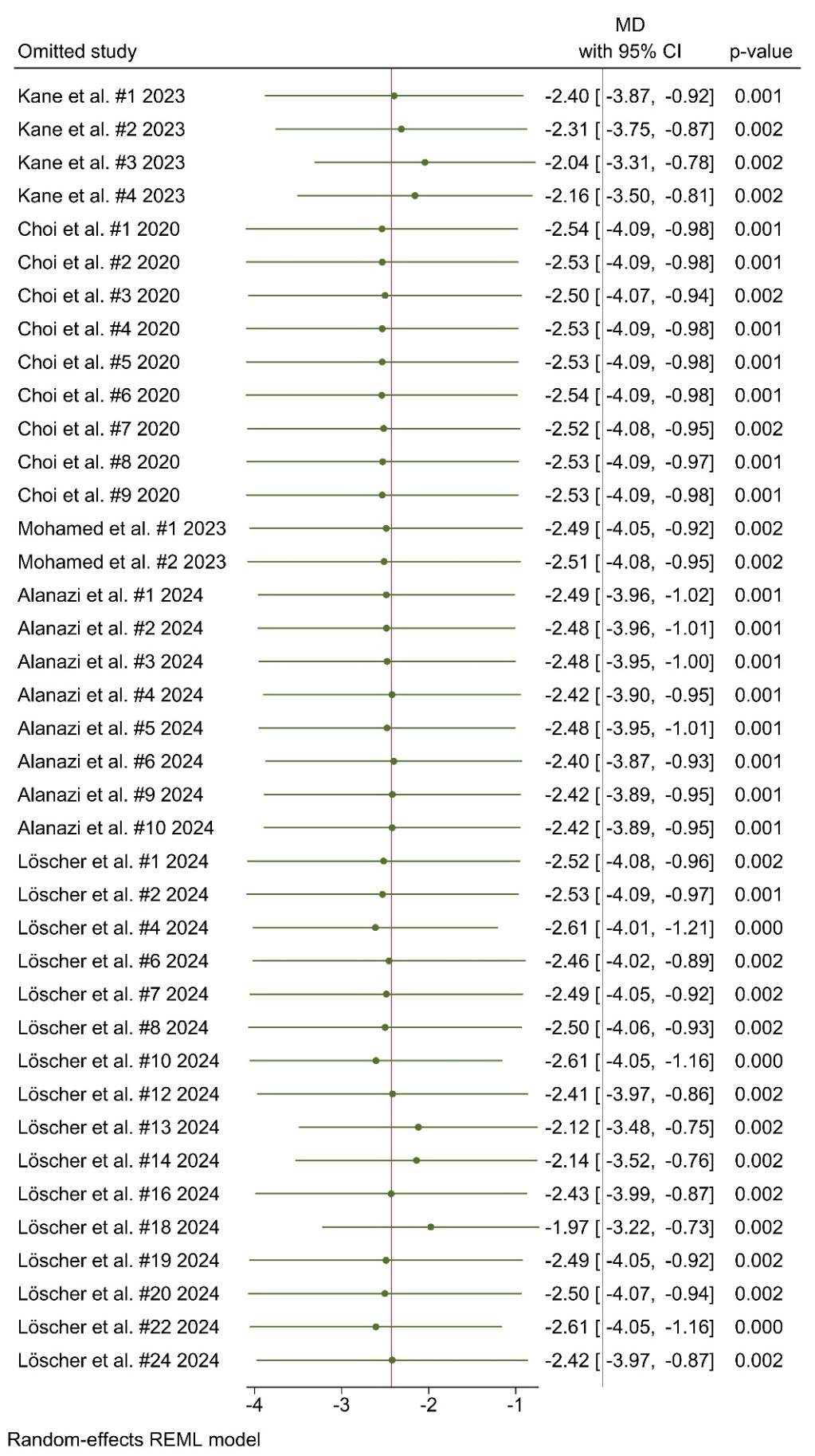


**Supplementary Figure 2.** Leave-one-out sensitivity analysis for tooth–base bonding comparisons after removing the outliers.
